# Cost of Public Health Insurance for US-Born and Immigrant Adults

**DOI:** 10.1001/jamanetworkopen.2023.34008

**Published:** 2023-09-15

**Authors:** Neeraj Kaushal, Felix M. Muchomba

**Affiliations:** 1 Columbia School of Social Work, Columbia University, New York, New York; 2 School of Social Work, Rutgers, The State University of New Jersey, New Brunswick

## Abstract

**Importance:**

The immigrant population in the US has low health insurance coverage. Surveys find that approximately one-half of the US population is opposed to public health insurance of immigrants, and there is a widely held belief that immigrants are a state fiscal liability.

**Objective:**

To estimate the cost of providing public health insurance to immigrants in the US.

**Design, Setting, and Participants:**

This serial cross-sectional study used restricted data from the 2011 to 2019 Medical Expenditure Panel Survey (and data from 2011-2020 in supplemental analyses). The data are nationally representative of the US civilian noninstitutionalized population. Participants included adults aged 19 to 64 years with family incomes below 138% of the Federal Poverty Level, the population that benefited from the Medicaid expansions. Data analysis was performed from November 2022 to August 2023.

**Exposures:**

State Medicaid expansion.

**Main Outcomes and Measures:**

The primary outcomes were insurance coverage, total health care expenditures, expenditures categorized by payment source (paid by self or family and paid by others), expenditures by major health care type (office based, inpatient, and prescription), and health care utilization (number of office-based visits, outpatient facility visits, emergency department visits, hospital discharges, dental care visits, home health clinician days, and prescription medicine refills). A difference-in-differences method was used to compare the health care cost and utilization by low-income, working-age US-born and immigrant adults in states that adopted the Patient Protection and Affordable Care Act (ACA) Medicaid expansions with the corresponding change in nonexpansion states before and after the policy implementation.

**Results:**

Among the study sample of 44 482 individuals (mean [SD] age, 38.5 [14.0] years; 25 221 female individuals [56.7%]; 34 052 [76.6%] US born), 46% of immigrant adults (1953 participants) and 70% of US-born adults (9396 participants) had insurance coverage in the pre-ACA period. Medicaid expansions increased insurance coverage of both groups by 7 percentage points (95% CI, 3 to 11 percentage points). The resulting change in health care increased total expenditures (self-paid plus insurer paid) by $660 (95% CI, $79 to $1242) and insurer-paid expenditures by $745 (95% CI, $141 to $1350) per US-born adult. For immigrant adults, the corresponding changes in total ($266; 95% CI, −$348 to $880) and insurer-paid ($308; 95% CI, −$352 to $968) expenditures were small and not statistically significant. Estimates suggest that providing insurance to immigrants costs the health care system approximately $3800 per person per year, less than one-half the corresponding cost ($9428 per person per year) for US-born adults.

**Conclusions and Relevance:**

These findings suggest that the direct cost of providing public health insurance to immigrants is less than that for the US born, and immigrants’ health care utilization, upon coverage, remains comparatively modest, thus refuting the notion that providing insurance to immigrants imposes a heavy fiscal burden.

## Key Points

**Question:**

What is the cost of providing public health insurance to immigrants in the US?

**Findings:**

This serial cross-sectional study of 44 482 low-income, working-age adults found that increased insurance coverage from the Medicaid expansion accompanying the Patient Protection and Affordable Care Act was associated with statistically significantly higher health care expenditures for US-born but not immigrant adults. Providing insurance to immigrants costs the health care system less than half the corresponding cost for US-born adults ($3800 vs $9428 per person per year).

**Meaning:**

These findings suggest that upon receiving public insurance, low-income immigrants’ health care utilization and cost remain low, whereas US-born adults’ health care utilization and costs increase, thus refuting the widely held belief that providing insurance to immigrants imposes a heavy fiscal burden.

## Teaser

This cross-sectional study estimates the cost of providing public health insurance to US-born adults compared with those who immigrate to the US.

## Introduction

The immigrant population in the US has low health insurance coverage, which impacts their health care use and, possibly, health.^1,2^ Yet, providing immigrants access to public health insurance is highly controversial. Opinion surveys show that approximately one-half the US population is opposed to public health insurance of immigrants.^3^ There is a widely held belief that immigrants are a state liability, and this belief has often influenced health policy.^4,5^ In 2018, for instance, the Trump administration changed the definition of public charge to include Medicaid and Child Health Insurance Program beneficiaries, thus discouraging immigrants from seeking public insurance even when eligible.^6,7,8^ Although the COVID-19 pandemic has revealed the risks of a policy that results in a substantial segment of the population without insurance, the question remains: what is the cost of providing public health insurance to immigrants and does it differ substantially from the corresponding cost for the US born?

In this article, we use restricted Medical Expenditure Panel Survey (MEPS) data for 2011 to 2019 (and 2011-2020 and 2011-2018 in supplemental analyses) to answer this question. An advantage of using MEPS is that it collects data on individuals’ out-of-pocket medical expenses, as well as total medical care expenses based on data from health care practitioners, which is essential to estimate the fiscal impact of public health insurance. Most public survey data report only out-of-pocket expenses of the surveyed population.

On average, compared with the US born, immigrants have healthy lifestyles, better health, and fewer preexisting conditions, and, consequently, lower health care utilization.^9,10,11,12,13,14,15^ Individuals with unhealthy lifestyles and poorer health are more likely to seek insurance.^16^ Because populations covered by insurance may be different from those not covered, simple comparisons of medical care cost of the 2 groups (uninsured vs insured) will likely have confounding bias, and such bias may be different among immigrant and US-born adults.^9,10,11,12,13,14^

We address this challenge by taking advantage of the increase in health insurance of immigrant and US-born populations resulting from Medicaid expansion accompanying the Patient Protection and Affordable Care Act (ACA). Notably, the ACA expanded Medicaid eligibility to adults with incomes below 138% of the Federal Poverty Level (FPL), but not all states complied with the federal law and those that did differed in their timing and degree of expansion. We use a difference-in-differences method comparing the change in medical care cost and utilization by low-income, working-age adults (aged 19-64 years) in states that adopted Medicaid expansion with the corresponding change in nonexpansion states before and after the ACA implementation. Our models adjust for base-level preexisting health conditions.

## Methods

### Data

MEPS is a nationally representative survey of health care utilization and spending for the civilian, noninstitutionalized US population.^17^ MEPS supplements information on health care expenditures from respondents with data from health care practitioners and institutions (ie, physicians, hospitals, home health agencies, and pharmacies). We used restricted-access MEPS data because information on respondents’ state of residence is not released publicly. Our analytic sample was adults aged 19 to 64 years with incomes below 138% of the FPL, the population that benefited from the Medicaid expansions. The Columbia University institutional review board deemed this research exempt from review and the need for informed consent because it is based on anonymized secondary data, in accordance with 45 CFR §46. This study follows the Strengthening the Reporting of Observational Studies in Epidemiology (STROBE) reporting guideline.

The outcomes of interest were whether respondents had any health insurance coverage during the calendar year, calendar-year total health care expenditures, expenditures categorized by payment source (paid by self or family and paid by others), expenditures on the 3 largest health care types (office-based, inpatient, and prescription), and 7 health care utilization outcomes (number of office-based visits, outpatient facility visits, emergency department visits, hospital discharges, dental care visits, home health clinician days, and prescription refills). We defined individuals who reported being born outside the US as immigrants.

We used information on diagnosed health conditions and age at diagnosis to control for whether individuals had a preexisting chronic condition (ie, received a diagnosis of stroke, emphysema, diabetes, arthritis, asthma, high blood pressure, or coronary heart disease ≥5 years prior). Information on age at diagnosis of cancer was not collected in all years. However, controlling for ever having had cancer did not change the results. We merged into the MEPS data state-year information on unemployment rate and per capita gross domestic product, which we controlled in our analysis. Sample and descriptive statistics are in eTable 1 in Supplement 1.

### Statistical Analysis

Data analysis was performed from November 2022 to August 2023. To identify associations of the ACA Medicaid expansion with insurance coverage and health expenditures, we used a difference-in-differences approach in which we compared changes in study outcomes among adults residing in Medicaid expansion states before and after the expansion vs those in states that did not expand. Although ACA Medicaid expansions were scheduled to start on January 1, 2014, some states expanded eligibility earlier, others expanded later, and some opted not to expand. Medicaid expansion states (and year of expansion if after 2014) include Alaska (2015), Arkansas, Arizona, California, Colorado, Connecticut, District of Columbia, Delaware, Hawaii, Iowa, Illinois, Indiana (2015), Kentucky, Louisiana (2016), Massachusetts, Maryland, Maine (2019), Michigan, Minnesota, Montana (2016), North Dakota, New Hampshire, New Jersey, New Mexico, Nevada, New York, Ohio, Oregon, Pennsylvania (2015), Rhode Island, Virginia (2019), Vermont, Washington, and West Virginia. In our main analyses, we created a binary state Medicaid expansion policy variable equal to 1 if the respondent resided in an expansion state and was interviewed after their state’s expansion date, otherwise 0. The Medicaid policy variable was set to 1 throughout for groups eligible for Medicaid over the entire study period (ie, all adults in Washington, DC, and Vermont and adults with children in the 7 states [Connecticut, Illinois, Minnesota, New Jersey, New York, Rhode Island, and Wisconsin] that had Medicaid income eligibility limits for parents above 138% of the FPL before 2014). The exclusion of Wisconsin, which was not an ACA expansion state but was categorized as one as in previous studies^18,19^ because it received federal approval under the Badgercare program to provide coverage to adults with incomes up to 100% of the FPL, did not affect the results.

We used multivariable linear models to estimate study outcomes (insurance, expenditures, and utilization) on the binary Medicaid policy variable. Expenditures were modeled using a 2-part model: first, we examined the probability that a person had any expenditures and then the logged conditional expenditures. We used Stata’s twopm command to compute overall marginal effects from the combined first-part and second-part models.^20^ Our analysis adjusted for age, self-reported race and ethnicity, sex, educational attainment, marital status, family size, number of children, presence of a preexisting chronic condition, and state and year fixed effects. Data on race and ethnicity were included to account for the cumulative impact of structural racism in the US. Because having insurance may affect the severity of a chronic condition and, thus, its reporting, we lagged the diagnosis of the chronic condition by 5 years. To account for any changes in state employment and economic output associated with state Medicaid expansion, we also controlled for state unemployment rate and state per capita gross domestic product. We examined whether effects of Medicaid expansion differed between US-born and immigrant adults by including in the models a variable on whether the respondent was an immigrant and an interaction between the Medicaid policy variable and immigrant status. We report robust SEs clustered on state of residence to account for arbitrary correlation of observations within each state. All analyses used MEPS sampling weights. We used complete case analysis because of low rates of missing data (<2% of our sample had any missing data). All analyses were performed using Stata/SE statistical software version 17 (StataCorp). Differences in health expenditures were assessed using a 2-sided test and a significance level of *P* < .05.

The difference-in-differences approach yields unbiased estimates of the outcomes of Medicaid expansion on the assumption that, after accounting for controls, the trend in health insurance and health care expenditures in expansion and nonexpansion states would have been similar if Medicaid expansion had not occurred. We examined this parallel-trends assumption using event study models for the key outcomes. Specifically, we used multivariable linear models for each outcome where a binary variable representing living in an expansion state was interacted with nativity and year indicator variables for all years except the year immediately before expansion (2013 for states in our main analyses). If the parallel trends assumption holds, the coefficients on these interaction terms should be statistically indistinguishable from 0 in the pre-ACA period.

## Results

**Figure 1.**
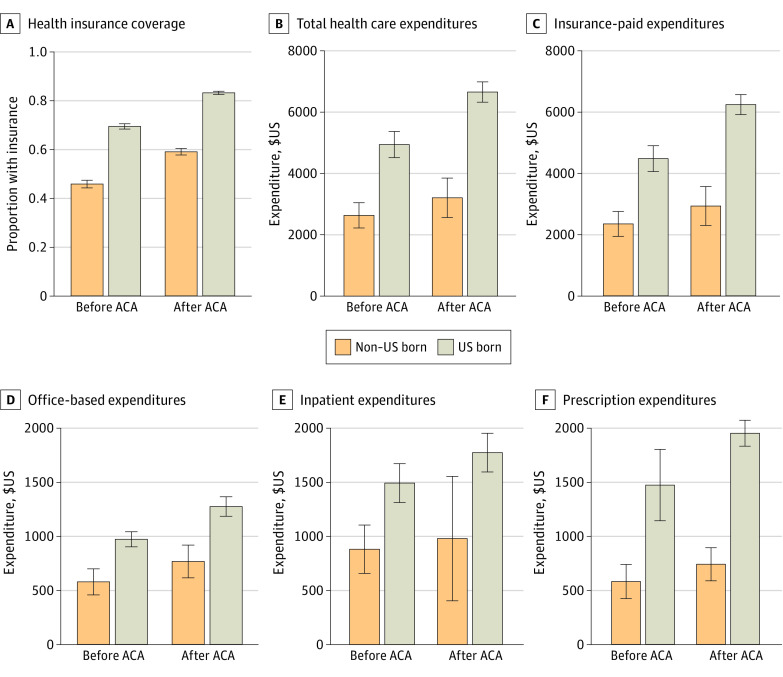
Health Insurance Coverage and Annual Health Care Expenditure by Place of Birth Graphs show data from before (2011-2013) and after (2014-2019) implementation of the Patient Protection and Affordable Care Act (ACA). The sample is restricted to adults aged 19 to 64 years in households with incomes below 138% of the Federal Poverty Level. Error bars are 95% CIs.

Among the study sample of 44 482 individuals (mean [SD] age, 38.5 [14.0] years; 25 221 female individuals [56.7%]; 34 052 [76.6%] US born), in the pre-ACA period, 46% of low-income, nonelderly, immigrant adults (1953 participants) and 70% of low-income US-born adults had insurance coverage (9396 participants) (Figure 1). Both groups experienced an increase in insurance coverage, but the gap between them remained large in the post-ACA period. In the pre-ACA period, health care expenditure on low-income immigrant adults (by self and insurer) was approximately one-half the corresponding amount on similar US-born adults. This could partly be on account of the lower insurance coverage of immigrants and partly due to differences in health, lifestyle (which may impact health care use), and obstacles to health care (eg, poorer English language proficiency, geographic inaccessibility to health care, or cultural obstacles) the 2 groups face. Indeed, descriptive statistics show that 36% of the US-born sample (4769 respondents) were obese compared with 28% of the immigrant sample (1136 respondents) in the pre-ACA data, and obesity levels were statistically the same in the post-ACA period. Furthermore, 44% of the US-born adult sample (15 043 respondents) had a preexisting chronic condition vs 26% of the immigrant sample (2663 respondents); adjusting for preexisting conditions decreased, but did not eliminate, the difference in health care expenditures between the 2 groups (eFigure 1 in Supplement 1).

As expected, after passage of the ACA, both groups experienced increases in medical expenditures and a decrease in the proportion of expenditures that was self-paid. However, the post-ACA gap in per capita health care expenditure between immigrant and US-born adults remained large.

We studied 3 specific expenditure categories—office-based, inpatient, and prescription drug expenditures—that accounted for 77% of the total expenditure in the preexpansion period. For all 3 categories, spending on immigrant adults was lower than that on US-born adults, and although expenses in all categories increased in the post-ACA period, here too, the gaps between the 2 groups remained large (Figure 1).

We first describe the outcome from the event study models as evidence of the validity of this method (eFigure 2 in Supplement 1). For 3 outcomes—whether the respondent has health insurance, total expenditure, and expenditure paid by other (primarily insurer)—the coefficients of the interaction between prepolicy years and states that expanded Medicaid were not statistically significant for either the US-born or immigrant sample, validating the assumption of parallel prepolicy trends. For self-paid or family-paid expenditures, for the US-born sample, the prepolicy interaction coefficients were sometimes statistically significant. Specifically, the results for the first part (binary outcome) were consistent with parallel trends, but those for the second part (conditional logged spending) were not always consistent with the assumption. This suggests that the parallel trend assumption was not valid (or weakly valid) for this outcome. Although we present the difference-in-difference estimates for this outcome, we do not discuss them as a cautionary measure.

eTable 2 in Supplement 1 shows a 7–percentage point (95% CI, 3 to 11 percentage points) increase in insurance coverage, and the estimated associations were statistically significantly the same for US-born and immigrant adults. This result is in line with studies that used earlier data on noncitizens (rather than all non–US-born adults) from the American Community Survey.^21,22^

**Figure 2.**
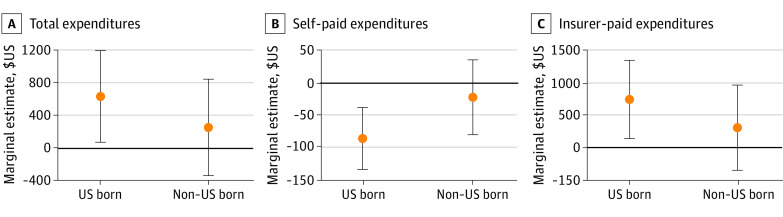
Association of State Medicaid Expansion With Annual Health Care Expenditures of US-Born and Immigrant Adults Aged 19-64 Years, 2011-2019 Graphs show marginal estimates and 95% CIs derived from 2-part models. Sample is restricted to adults aged 19 to 64 years in households with incomes below 138% of the Federal Poverty Level. All models control for individual-level age, sex, race and ethnicity, educational attainment, marital status, family size, number of children, preexisting chronic condition, state unemployment rate, state gross domestic product, state fixed effects, and year fixed effects. Robust SEs are clustered on state of residence. Results are adjusted for Medical Expenditure Panel Survey sampling weights.

Estimates in Figure 2 suggest that Medicaid expansions were associated with a statistically significant increase ($660; 95% CI, $79 to $1242; or 11%) in expenditure for US-born individuals and a small but not statistically significant increase ($266; 95% CI, −$348 to $880; or 9%) in expenditure for the immigrant population. Results for underlying 2-part models are in eTable 2 in Supplement 1. We also found an increase in expenditures paid by others (insurer expenditure) of $745 (95% CI, $141 to $1350, or 13%) for the US born and a not statistically significant increase of $308 (95% CI, −$352 to $968) for the immigrant population (Figure 2).

**Figure 3.**
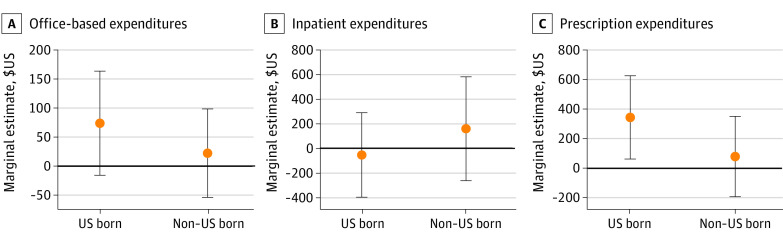
Association of State Medicaid Expansion With Annual Health Care Expenditures, in Major Categories, of US-Born and Immigrant Adults Aged 19-64 Years, 2011-2019 Graphs show marginal estimates and 95% CIs derived from 2-part models. See the Figure 2 caption for sample and model specifications. Results are for the 3 largest expenditure categories, which account for 77% of the total health care expenditure.

Figure 3 presents marginal effect estimates for the 3 largest expenditure categories. Results for underlying 2-part models are in eTable 3 in Supplement 1. There are 2 points to note: One, we found a 5–percentage point increase in adults incurring any office-based practitioner expenses, no change in proportion with positive inpatient expenditures, and a 2–percentage point increase in adults incurring prescription expenditure; the estimated coefficients were the same for immigrant and US-born individuals (eTable 3 in Supplement 1). Two, marginal estimates from the combined 2-part models suggest a $347 increase in prescription expenditure by US-born individuals, but a nonsignificant change for immigrants (Figure 3).

Next, we examine 7 specific indicators and categories of health care utilization, and the results are shown in the Table and eTables 4 and 5 in Supplement 1. Medicaid expansions were associated with increases in office visits, outpatient facility visits, dental visits, and prescription refills by US-born adults. Estimates of the coefficient on the interaction term between state and Medicaid policy for immigrant individuals were negative and statistically significant for outpatient facility visits and not significant for other outcomes, but often were estimated with large SEs.

**Table.** Association of State Medicaid Expansion With Health Care Utilization of US-Born and Immigrant Adults Aged 19-64 Years, 2011-2019

Independent variables	Dependent variables, mean (SE), estimate^a^						
	Office-based visits (n = 43 961)	Outpatient facility visits (n = 43 961)	Emergency department visits (n = 43 961)	Hospital discharges (n = 43 961)	Dental care visits (n = 43 961)	Home health practitioner days (n = 43 848)	Prescription medicine refills (n = 43 961)
State Medicaid expansion	0.314 (0.089)^b^	0.184 (0.088)^c^	−0.010 (0.057)	0.034 (0.038)	0.178 (0.055)^b^	0.080 (0.046)	0.241 (0.070)^b^
Immigrant	−0.674 (0.061)^b^	−0.143 (0.051)^b^	−0.486 (0.045)^b^	−0.201 (0.027)^b^	−0.165 (0.058)^b^	−0.099 (0.031)^b^	−0.929 (0.083)^b^
State Medicaid expansion × immigrant	−0.132 (0.074)	−0.202 (0.066)^b^	0.085 (0.061)	0.024 (0.050)	0.031 (0.071)	−0.068 (0.053)	−0.104 (0.084)
Pre-ACA mean of outcome, No.	4.897	0.399	0.349	0.135	0.441	2.476	13.140

Abbreviation: ACA, Patient Protection and Affordable Care Act.

^a^ Dependent variables are log-transformed in the regression analysis. See the Figure 2 caption for sample restriction and model specification. Robust SEs were clustered on state of residence.

^b^ *P* < .01.

^c^ *P* < .05.

In sensitivity analyses, we conducted 4 specification checks to test the validity of our main analysis. First, because the federal policy does not allow immigrants who are in the US for less than 5 years access to public health insurance, we estimated models restricting the immigrant sample to respondents who were in the US for 5 years or longer. We also estimated models using state-level differences in policy for those in the US for less than 5 years. The results, shown in eTables 6, 7, 8, and 9 in Supplement 1, were similar to those shown in Figure 2 and Figure 3 and eTables 2 and 3 in Supplement 1.

Second, the difference-in-differences analysis is likely to yield biased estimates if the policy is staggered.^23^ In addition to event study estimates, we did an analysis where we defined as expansion states those that expanded Medicaid in early 2014 and defined as nonexpansion states those that did not expand Medicaid or expanded after December 2019, and excluded all other states. Estimates were similar to the main results (eTables 10 and 11 in Supplement 1).

Third, marketplace subsidies associated with ACA, such as Premium Tax Credits and Cost-Sharing Reduction, increased insurance coverage for adults with incomes 100% to 400% of the FPL, particularly in nonexpansion states.^24,25^ To test whether market subsidies associated with ACA impacted the outcome of our analysis, we also estimated models restricting sample to those with household incomes less than 100% of the FPL. Estimates were similar to the main results (eTables 12 and 13 in Supplement 1). Furthermore, controlling for respondent’s employment yielded similar results (eTable 14 in Supplement 1).

Finally, we estimated models using (1) 2011 to 2018 data to avoid bias on account of changes in immigrant eligibility under the US Department of Homeland Security definition of public charge directive, and (2) 2011 to 2020 data to check whether our findings hold after adding the most recent year for which MEPS data are available. For each set, we estimated models for populations below 138% of the FPL and 100% of the FPL. The results for the first model are in eTable 15 in Supplement 1 (2011-2018) and are substantially the same as our primary results using data for 2011 to 2019. The results for the second model are in eTable 16 in Supplement 1. Here, estimates are similar to those using the 2011 to 2019 data with one difference: the estimated association of Medicaid expansions with total expenditures was not statistically significant. However, as in the earlier analysis, Medicaid expansions were associated with decreased self-paid expenditures and increased insurer-paid expenditures of the US born but were not associated with these outcomes for the immigrant population.

## Discussion

In this cross-sectional study, we found that the ACA Medicaid expansions were associated with similar, statistically significant increases in insurance coverage of low-income, working-age, US-born and immigrant adults, but the associations with health care expenditure and health care utilization differed between the 2 groups. Among the US born, Medicaid expansion increased total health care expenditure by 11% ($660 per person per year) and insurer-paid expenditures by 13% ($745). The corresponding changes in expenditures for immigrants were negligible and not statistically significant.

Assuming that all of the increase in cost is associated with the increase in insurance coverage, our calculations show that the direct cost (self + insurer) of health insurance to an additional immigrant nonelderly adult is approximately $3800 per person per year (ie, the coefficient on marginal effect on expenditure / coefficient on change in insurance coverage = $266/0.07), which is less than half the corresponding cost ($660/0.07 = $9428) of insurance to a US-born nonelderly adult.

We also found that Medicaid expansions were associated with increased office-based practitioner visits and dental visits of both groups and increases in out-patient facility visits and prescription medicine refills by US-born adults, but not by immigrants. Results controlled for preexisting conditions and remained robust to a large set of sensitivity checks, including restricting the analysis to respondents with incomes less than 100% of the FPL and restricting analyses to immigrants in the US for more than 5 years. Estimates remain robust when we restricted the data to 2011 to 2018 and expanded to 2011 to 2020.

Efforts to increase coverage of low-income immigrants by extending public health insurance have been stymied by health policy founded on the belief that such action would be a large fiscal burden for state and federal governments, even though these beliefs are not backed by actual data.^26,27^ Instead, our study indicates that public health insurance for immigrants increased their coverage; the concomitant increases in health care costs per capita and utilization were much smaller for immigrants than for the US-born population. Our results add to previous research^26,27,28^ based on private insurance and Medicare populations that found immigrants’ contributions to the health care system exceeded their health care costs.

Arguably, the differential response from the 2 groups could be attributed to unobserved health, behavior, and cultural differences between them.^15,29^ It is also possible that some immigrants in our sample hesitate to seek public health care, even when eligible, for fear of jeopardizing their future immigration status or those of their family members.

### Limitations

There are limitations to our study. First, our data are not able to distinguish between documented and undocumented immigrants. To address this concern, in a supplemental analysis, we merged our MEPS data with data on the same respondents from the National Health Interview Survey that has information on citizenship status. In that analysis, we found that our conclusions hold even for immigrant US citizens. Second, the data do not include institutionalized groups (eg, nursing home residents), which may bias downward the health expenditures. Our results may be conservative on account of this limitation because a majority of people in institutional care are elderly, and the immigrant population is relatively young. Third, although the immigrant population is highly diverse, we did not study differences in health care expenditures across immigrant groups, which is an important topic for future research.^14,30^

## Conclusions

The immigrant population in the US has low health insurance that impacts their health care use and possibly health. Yet, concerns about the fiscal ramification of providing public health insurance to immigrants remain strong. Much of the public debate on the issue lacks rigorous systematic evidence of the effect of health insurance on health care costs of immigrants. Our study bridges this gap and shows that the total medical care expenditure (out-of-pocket and insurance covered) of low-income, immigrant, working-age adults in the US is less than one-half the corresponding expenditure of similar US-born adults.

## Notes

Supplement 1. **eTable 1.** Sample Characteristics by Nativity and Pre/Post ACA **eFigure 1.** Health Insurance Coverage and Annual Healthcare Expenditure by Nativity in the Pre-ACA (2011-2013) and Post-ACA (2014-2019) Periods, Adjusted for Whether Has Preexisting Chronic Condition **eFigure 2.** Coefficient Estimates and 95% Confidence Intervals From Event Study of Medicaid Expansion by Nativity Status **eTable 2.** Estimates of the Effect of State Medicaid Expansion on Health Insurance Coverage and Annual Healthcare Expenditures of US-Born and Foreign-Born Adults Aged 19-64, With Incomes Below 138% of the Federal Poverty Threshold, 2011-2019 **eTable 3.** Estimates of the Effect of State Medicaid Expansion on Annual Healthcare Expenditures, by Healthcare Type, of US-Born and Foreign-Born Adults Aged 19-64, 2011-2019 **eTable 4.** Effect of State Medicaid Expansion on Healthcare Utilization of US-Born and Foreign-Born Adults Aged 19-64, 2011-2019 **eTable 5.** Effect of State Medicaid Expansion on (Dichotomized) Healthcare Utilization of US-Born and Foreign-Born Adults Aged 19-64, 2011-2019 **eTable 6.** Estimates of the Effect of State Medicaid Expansion on Health Insurance Coverage and Annual Healthcare Expenditures of US-Born and Foreign-Born Adults in the US for 5+ Years Aged 19-64, 2011-2019 **eTable 7.** Estimates of the Effect of State Medicaid Expansion on Annual Healthcare Expenditures, by Healthcare Type, of US-Born Adults and Foreign-Born Adults in the US for 5+ Years Aged 19-64, 2011-2019 **eTable 8.** Estimates of the Effect of State Medicaid Expansion on Health Insurance Coverage and Annual Healthcare Expenditures of US-Born and Foreign-Born Adults (Alternate Specification Using State-Level Differences in Medicaid Eligibility for Foreign-Born Adults in the US for <5 Years), 2011-2019 **eTable 9.** Estimates of the Effect of State Medicaid Expansion on Annual Healthcare Expenditures, by Healthcare Type, of US-Born Adults and Foreign-Born Adults (Alternate Specification: Using State-Level Differences in Medicaid Eligibility for Foreign-Born Adults in the US for <5 Years), 2011-2019 **eTable 10.** Estimates of the Effect of State Medicaid Expansion on Health Insurance Coverage and Annual Healthcare Expenditures of US-Born and Foreign-Born Adults Aged 19-64, Excluding Early and Late Expansion States, 2011-2019 **eTable 11.** Estimates of the Effect of State Medicaid Expansion on Annual Healthcare Expenditures, by Healthcare Type, of US-Born and Foreign-Born Adults Aged 19-64, Excluding Early and Late Expansion States, 2011-2019 **eTable 12.** Estimates of the Effect of State Medicaid Expansion on Health Insurance Coverage and Annual Healthcare Expenditures of US-Born and Foreign-Born Adults Aged 19-64 With Incomes Below 100% of FPL, 2011-2019 **eTable 13.** Estimates of the Effect of State Medicaid Expansion on Annual Healthcare Expenditures, by Healthcare Type, of US-Born and Foreign-Born Adults Aged 19-64 With Incomes Below 100% of FPL, 2011-2019 **eTable 14.** Estimates of the Effect of State Medicaid Expansion on Health Insurance Coverage and Annual Healthcare Expenditures of US-Born and Foreign-Born Adults Aged 19-64, With Additional Adjustment for Employment Status, 2011-2019 **eTable 15.** Estimates of the Effect of State Medicaid Expansion on Health Insurance Coverage and Annual Healthcare Expenditures of US-Born and Foreign-Born Adults Aged 19-64 Using MEPS 2011-2018 **eTable 16.** Estimates of the Effect of State Medicaid Expansion on Health Insurance Coverage and Annual Healthcare Expenditures of US-Born and Foreign-Born Adults Aged 19-64 Using MEPS 2011-2020

Supplement 2.
Data Sharing Statement

## References

[1] Capps R, Fix M. Immigration reform: a long road to citizenship and insurance coverage. Health Aff (Millwood). 2013;32(4):639-642. doi:10.1377/hlthaff.2013.0187 23569042

[2] Kaiser Family Foundation. Health coverage of immigrants. December 20, 2022. Accessed July 29, 2023. https://www.kff.org/racial-equity-and-health-policy/fact-sheet/health-coverage-of-immigrants/

[3] Pew Research Center for the People & the Press, Pew Hispanic Center. No consensus on immigration problem or proposed fixes: America’s immigration quandary. March 30, 2006. Accessed July 29, 2022. https://www.pewresearch.org/wp-content/uploads/sites/5/reports/63.pdf

[4] Camarota SA; Center for Immigration Studies. Welfare use by immigrant and native households: an analysis of Medicaid, cash, food, and housing programs. September 2015. Accessed July 29, 2022. https://cis.org/sites/default/files/camarota-welfare-final.pdf

[5] Edwards JR; Center for Immigration Studies. The Medicaid costs of legalizing illegal aliens. July 12, 2010. Accessed July 29, 2022. https://cis.org/Medicaid-Costs-Legalizing-Illegal-Aliens

[6] Parmet WE. The Trump administration’s new public charge rule: implications for health care & public health. Health Affairs Blog. August 13, 2019. Accessed August 16, 2022. https://www.healthaffairs.org/content/forefront/trump-administration-s-new-public-charge-rule-implications-health-care-public-health

[7] Department of Homeland Security. Inadmissibility on public charge grounds. August 14, 2019. Accessed July 29, 2022. https://www.govinfo.gov/content/pkg/FR-2019-08-14/pdf/2019-17142.pdf

[8] Calvo JM. Trump order mandating deportation for health service use: not legally sufficient. Am J Public Health. 2017;107(8):1240-1241. doi:10.2105/AJPH.2017.303896 28700292 PMC5508167

[9] Kaushal N, Kaestner R. Welfare reform and health insurance of immigrants. Health Serv Res. 2005;40(3):697-721. doi:10.1111/j.1475-6773.2005.00381.x 15960687 PMC1361164

[10] Fix M, Haskins R; The Brookings Institution. Welfare benefits for non-citizens. February 2, 2002. Accessed July 29, 2022. https://www.brookings.edu/research/welfare-benefits-for-non-citizens/

[11] Mohanty SA, Woolhandler S, Himmelstein DU, Pati S, Carrasquillo O, Bor DH. Health care expenditures of immigrants in the United States: a nationally representative analysis. Am J Public Health. 2005;95(8):1431-1438. doi:10.2105/AJPH.2004.044602 16043671 PMC1449377

[12] Derose KP, Bahney BW, Lurie N, Escarce JJ. Review: immigrants and health care access, quality, and cost. Med Care Res Rev. 2009;66(4):355-408. doi:10.1177/1077558708330425 19179539

[13] Wilson FA, Zallman L, Pagán JA, . Comparison of use of health care services and spending for unauthorized immigrants vs authorized immigrants or US citizens using a machine learning model. JAMA Netw Open. 2020;3(12):e2029230. doi:10.1001/jamanetworkopen.2020.29230 33306118 PMC7733155

[14] Flavin L, Zallman L, McCormick D, Wesley Boyd J. Medical expenditures on and by immigrant populations in the United States. Int J Health Serv. 2018;48(4):601-621. doi:10.1177/0020731418791963 30088434

[15] Neuman S. Are immigrants healthier than native residents? IZA World Labor. 2014;108. doi:10.15185/izawol.108

[16] Hackmann MB, Kolstad JT, Kowalski AE. Health reform, health insurance, and selection: estimating selection into health insurance using the Massachusetts health reform. Am Econ Rev. 2012;102(3):498-501. doi:10.1257/aer.102.3.498 26321764 PMC4551461

[17] Chowdhury SR, Machlin SR, Gwet KL; Agency for Healthcare Research and Quality. Sample designs of the medical expenditure panel survey household component, 1996-2006 and 2007-2016. January 2019. Accessed July 29, 2022. https://meps.ahrq.gov/data_files/publications/mr33/mr33.pdf

[18] Simon K, Soni A, Cawley J. The impact of health insurance on preventive care and health behaviors: evidence from the first two years of the ACA Medicaid expansions. J Policy Anal Manage. 2017;36(2):390-417. doi:10.1002/pam.21972 28378959

[19] Soni A, Burns ME, Dague L, Simon KI. Medicaid expansion and state trends in Supplemental Security Income program participation. Health Aff (Millwood). 2017;36(8):1485-1488. doi:10.1377/hlthaff.2016.1632 28784742

[20] Belotti F, Deb P, Manning WG, Norton EC. Twopm: two-part models. Stata J. 2015;15(1):3-20. doi:10.1177/1536867X1501500102

[21] Stimpson JP, Wilson FA. Medicaid expansion improved health insurance coverage for immigrants, but disparities persist. Health Aff (Millwood). 2018;37(10):1656-1662. doi:10.1377/hlthaff.2018.0181 30273021

[22] Guo H, Zou M. Do non-citizens migrate for welfare benefits? evidence from the Affordable Care Act Medicaid expansion. Front Public Health. 2022;10:955257. doi:10.3389/fpubh.2022.955257 36249197 PMC9562776

[23] Callaway B, Sant’Anna PHC. Difference-in-differences with multiple time periods. J Econom. 2021;225(2):200-230. doi:10.1016/j.jeconom.2020.12.001

[24] Kaiser Family Foundation. Marketplace effectuated enrollment and financial assistance. 2022. Accessed September 30, 2022. https://www.kff.org/other/state-indicator/effectuated-marketplace-enrollment-and-financial-assistance/

[25] Kaiser Family Foundation. Explaining health care reform: questions about health insurance subsidies. November 2018. Accessed September 30, 2022. https://files.kff.org/attachment/Issue-Brief-Explaining-Health-Care-Reform-Questions-about-Health-Insurance-Subsidies

[26] Zallman L, Woolhandler S, Touw S, Himmelstein DU, Finnegan KE. Immigrants pay more in private insurance premiums than they receive in benefits. Health Aff (Millwood). 2018;37(10):1663-1668. doi:10.1377/hlthaff.2018.0309 30273017

[27] Zallman L, Wilson FA, Stimpson JP, . Unauthorized immigrants prolong the life of Medicare’s trust fund. J Gen Intern Med. 2016;31(1):122-127. doi:10.1007/s11606-015-3418-z 26084972 PMC4699990

[28] Zallman L, Woolhandler S, Himmelstein D, Bor D, McCormick D. Immigrants contributed an estimated $115.2 billion more to the Medicare Trust Fund than they took out in 2002-09. Health Aff (Millwood). 2013;32(6):1153-1160. doi:10.1377/hlthaff.2012.1223 23720486

[29] Singh GK, Siahpush M. Ethnic-immigrant differentials in health behaviors, morbidity, and cause-specific mortality in the United States: an analysis of two national data bases. Hum Biol. 2002;74(1):83-109. doi:10.1353/hub.2002.0011 11931581

[30] Sarría-Santamera A, Hijas-Gómez AI, Carmona R, Gimeno-Feliú LA. A systematic review of the use of health services by immigrants and native populations. Public Health Rev. 2016;37(1):28. doi:10.1186/s40985-016-0042-3 29450069 PMC5810113

