# Supplementary material for: Cost of Public Health Insurance for US-Born and Immigrant Adults
Source: JAMA Netw Open. 2023 Sep 15;6(9):e2334008. doi: 10.1001/jamanetworkopen.2023.34008 (PMC10504616; doi:10.1001/jamanetworkopen.2023.34008)
Supplement: Supplement 1. — eTable 1. Sample Characteristics by Nativity and Pre/Post ACA eFigure 1. Health Insurance Coverage and Annual Healthcare Expenditure by Nativity in the Pre-ACA (2011-2013) and Post-ACA (2014-2019) Periods, Adjusted for Whether Has Preexisting Chronic Condition eFigure 2. Coefficient Estimates and 95% Confidence Intervals From Event Study of Medicaid Expansion by Nativity Status eTable 2. Estimates of the Effect of State Medicaid Expansion on Health Insurance Coverage and Annual Healthcare Expenditures of US-Born and Foreign-Born Adults Aged 19-64, With Incomes Below 138% of the Federal Poverty Threshold, 2011-2019 eTable 3. Estimates of the Effect of State Medicaid Expansion on Annual Healthcare Expenditures, by Healthcare Type, of US-Born and Foreign-Born Adults Aged 19-64, 2011-2019 eTable 4. Effect of State Medicaid Expansion on Healthcare Utilization of US-Born and Foreign-Born Adults Aged 19-64, 2011-2019 eTable 5. Effect of State Medicaid Expansion on (Dichotomized) Healthcare Utilization of US-Born and Foreign-Born Adults Aged 19-64, 2011-2019 eTable 6. Estimates of the Effect of State Medicaid Expansion on Health Insurance Coverage and Annual Healthcare Expenditures of US-Born and Foreign-Born Adults in the US for 5+ Years Aged 19-64, 2011-2019 eTable 7. Estimates of the Effect of State Medicaid Expansion on Annual Healthcare Expenditures, by Healthcare Type, of US-Born Adults and Foreign-Born Adults in the US for 5+ Years Aged 19-64, 2011-2019 eTable 8. Estimates of the Effect of State Medicaid Expansion on Health Insurance Coverage and Annual Healthcare Expenditures of US-Born and Foreign-Born Adults (Alternate Specification Using State-Level Differences in Medicaid Eligibility for Foreign-Born Adults in the US for <5 Years), 2011-2019 eTable 9. Estimates of the Effect of State Medicaid Expansion on Annual Healthcare Expenditures, by Healthcare Type, of US-Born Adults and Foreign-Born Adults (Alternate Specification: Using State-Level Differences in Medica [file jamanetwopen-e2334008-s001.pdf]

Supplemental Online Content

Kaushal N, Muchomba FM. Cost of public health insurance for US-born and immigrant adults. *JAMA Netw Open*. 2023;6(9):e2334008.
doi:10.1001/jamanetworkopen.2023.34008

eTable 1. Sample Characteristics by Nativity and Pre/Post ACA
eFigure 1. Health Insurance Coverage and Annual Healthcare Expenditure by Nativity in the Pre-ACA (2011-2013) and Post-ACA (2014-2019) Periods, Adjusted for Whether Has Preexisting Chronic Condition
eFigure 2. Coefficient Estimates and 95% Confidence Intervals From Event Study of Medicaid Expansion by Nativity Status
eTable 2. Estimates of the Effect of State Medicaid Expansion on Health Insurance Coverage and Annual Healthcare Expenditures of US-Born and Foreign-Born Adults Aged 19-64, With Incomes Below 138% of the Federal Poverty Threshold, 2011-2019
eTable 3. Estimates of the Effect of State Medicaid Expansion on Annual Healthcare Expenditures, by Healthcare Type, of US-Born and Foreign-Born Adults Aged 19-64, 2011-2019
eTable 4. Effect of State Medicaid Expansion on Healthcare Utilization of US-Born and Foreign-Born Adults Aged 19-64, 2011-2019
eTable 5. Effect of State Medicaid Expansion on (Dichotomized) Healthcare Utilization of US-Born and Foreign-Born Adults Aged 19-64, 2011-2019
eTable 6. Estimates of the Effect of State Medicaid Expansion on Health Insurance Coverage and Annual Healthcare Expenditures of US-Born and Foreign-Born Adults in the US for 5+ Years Aged 19-64, 2011-2019
eTable 7. Estimates of the Effect of State Medicaid Expansion on Annual Healthcare Expenditures, by Healthcare Type, of US-Born Adults and Foreign-Born Adults in the US for 5+ Years Aged 19-64, 2011-2019
eTable 8. Estimates of the Effect of State Medicaid Expansion on Health Insurance Coverage and Annual Healthcare Expenditures of US-Born and Foreign-Born Adults (Alternate Specification Using State-Level Differences in Medicaid Eligibility for Foreign-Born Adults in the US for <5 Years), 2011-2019
eTable 9. Estimates of the Effect of State Medicaid Expansion on Annual Healthcare Expenditures, by Healthcare Type, of US-Born Adults and Foreign-Born Adults (Alternate Specification: Using State-Level Differences in Medicaid Eligibility for Foreign-Born Adults in the US for <5 Years), 2011-2019
eTable 10. Estimates of the Effect of State Medicaid Expansion on Health Insurance Coverage and Annual Healthcare Expenditures of US-Born and Foreign-Born Adults Aged 19-64, Excluding Early and Late Expansion States, 2011-2019
eTable 11. Estimates of the Effect of State Medicaid Expansion on Annual Healthcare Expenditures, by Healthcare Type, of US-Born and Foreign-Born Adults Aged 19-64, Excluding Early and Late Expansion States, 2011-2019
eTable 12. Estimates of the Effect of State Medicaid Expansion on Health Insurance Coverage and Annual Healthcare Expenditures of US-Born and Foreign-Born Adults Aged 19-64 With Incomes Below 100% of FPL, 2011-2019

© 2023 Kaushal N et al. *JAMA Network Open.*

eTable 13. Estimates of the Effect of State Medicaid Expansion on Annual Healthcare Expenditures, by Healthcare Type, of US-Born and Foreign-Born Adults Aged 19-64 With Incomes Below 100% of FPL, 2011-2019
eTable 14. Estimates of the Effect of State Medicaid Expansion on Health Insurance Coverage and Annual Healthcare Expenditures of US-Born and Foreign-Born Adults Aged 19-64, With Additional Adjustment for Employment Status, 2011-2019
eTable 15. Estimates of the Effect of State Medicaid Expansion on Health Insurance Coverage and Annual Healthcare Expenditures of US-Born and Foreign-Born Adults Aged 19-64 Using MEPS 2011-2018
eTable 16. Estimates of the Effect of State Medicaid Expansion on Health Insurance Coverage and Annual Healthcare Expenditures of US-Born and Foreign-Born Adults Aged 19-64 Using MEPS 2011-2020

This supplemental material has been provided by the authors to give readers additional information about their work.

© 2023 Kaushal N et al. *JAMA Network Open.*

eTable 1. Sample Characteristics by Nativity and Pre/Post ACA

	All		US born		Foreign born		Pre-ACA 2011-2013		Post-ACA 2014-2019	
	mean	se	mean	se	mean	se	mean	se	mean	se
Had any insurance	0.729	0.003	0.786	0.003	0.544	0.005	0.640	0.005	0.779	0.003
Total health care expenditure ($s)	5,294	114	6,004	133	2,975	220	4,361	172	5,818	150
Health care expenditure paid by self/family ($s)	375	9	411	11	256	14	400	15	361	11
Health care expenditure paid by others ($s)	4,919	113	5,593	131	2,719	218	3,960	170	5,458	147
Had any health care expenditure	0.751	0.003	0.792	0.003	0.617	0.005	0.740	0.004	0.758	0.003
Had health care expenditure paid by self/family	0.626	0.003	0.664	0.003	0.502	0.005	0.639	0.005	0.619	0.004
Had health care expenditure paid by others	0.679	0.003	0.728	0.003	0.519	0.005	0.643	0.005	0.699	0.004
Age	38.481	0.090	38.020	0.110	39.984	0.139	38.083	0.144	38.703	0.116
Male	0.433	0.003	0.431	0.004	0.439	0.005	0.439	0.005	0.430	0.004
Hispanic	0.255	0.002	0.127	0.002	0.674	0.005	0.257	0.004	0.254	0.003
Non-Hispanic Asian	0.049	0.001	0.013	0.001	0.164	0.004	0.044	0.002	0.051	0.002
Non-Hispanic Black	0.202	0.002	0.240	0.003	0.075	0.003	0.203	0.003	0.201	0.003
Non-Hispanic Other	0.038	0.001	0.047	0.002	0.009	0.001	0.029	0.002	0.043	0.002
Non-Hispanic White	0.456	0.003	0.572	0.004	0.078	0.004	0.466	0.005	0.451	0.004
Married	0.299	0.003	0.235	0.003	0.505	0.005	0.311	0.005	0.292	0.004
Widowed	0.029	0.001	0.031	0.001	0.021	0.001	0.025	0.002	0.031	0.001
Divorced	0.153	0.002	0.171	0.003	0.093	0.003	0.156	0.004	0.151	0.003
Separated	0.051	0.001	0.049	0.002	0.056	0.002	0.052	0.002	0.050	0.002
Never Married	0.469	0.003	0.513	0.004	0.324	0.005	0.456	0.005	0.475	0.004
Less than high school	0.275	0.003	0.219	0.003	0.459	0.005	0.295	0.004	0.263	0.003
High school or GED	0.364	0.003	0.393	0.004	0.267	0.005	0.343	0.005	0.376	0.004
Some college	0.201	0.003	0.226	0.003	0.119	0.004	0.229	0.004	0.186	0.003
Associate’s degree	0.064	0.002	0.072	0.002	0.040	0.002	0.044	0.002	0.076	0.002
Bachelor’s degree or higher	0.096	0.002	0.090	0.002	0.114	0.004	0.089	0.003	0.099	0.003
Foreign born	0.234	0.002	0.000	0.000	1.000	0.000	0.240	0.004	0.232	0.003
Has a pre-existing chronic condition	0.398	0.003	0.442	0.004	0.256	0.005	0.376	0.005	0.411	0.004
Number of children	0.900	0.008	0.756	0.009	1.370	0.015	0.910	0.012	0.894	0.010
Family size	3.011	0.011	2.744	0.012	3.882	0.021	3.034	0.018	2.998	0.014
Has obesity (body mass index ≥ 30 kg/m²)	0.350	0.003	0.367	0.004	0.293	0.005	0.345	0.005	0.354	0.005
30mins moderate/vigorous exercise 3x/week	0.469	0.003	0.486	0.004	0.412	0.005	0.468	0.005	0.469	0.004
N	44,482		34,052		10,430		15,992		28,490	

© 2023 Kaushal N et al. *JAMA Network Open.*

Note: se=Standard error. Non-Hispanic Other included individuals who identified as American Indian or Alaska Native, Native Hawaiian, Guamanian or Chamorro, Samoan, or other Pacific Islander, or who specified any other racial group that was not Asian, Black, or White. Includes 521 participants with some missing data who were excluded from regression analyses.

© 2023 Kaushal N et al. *JAMA Network Open.*

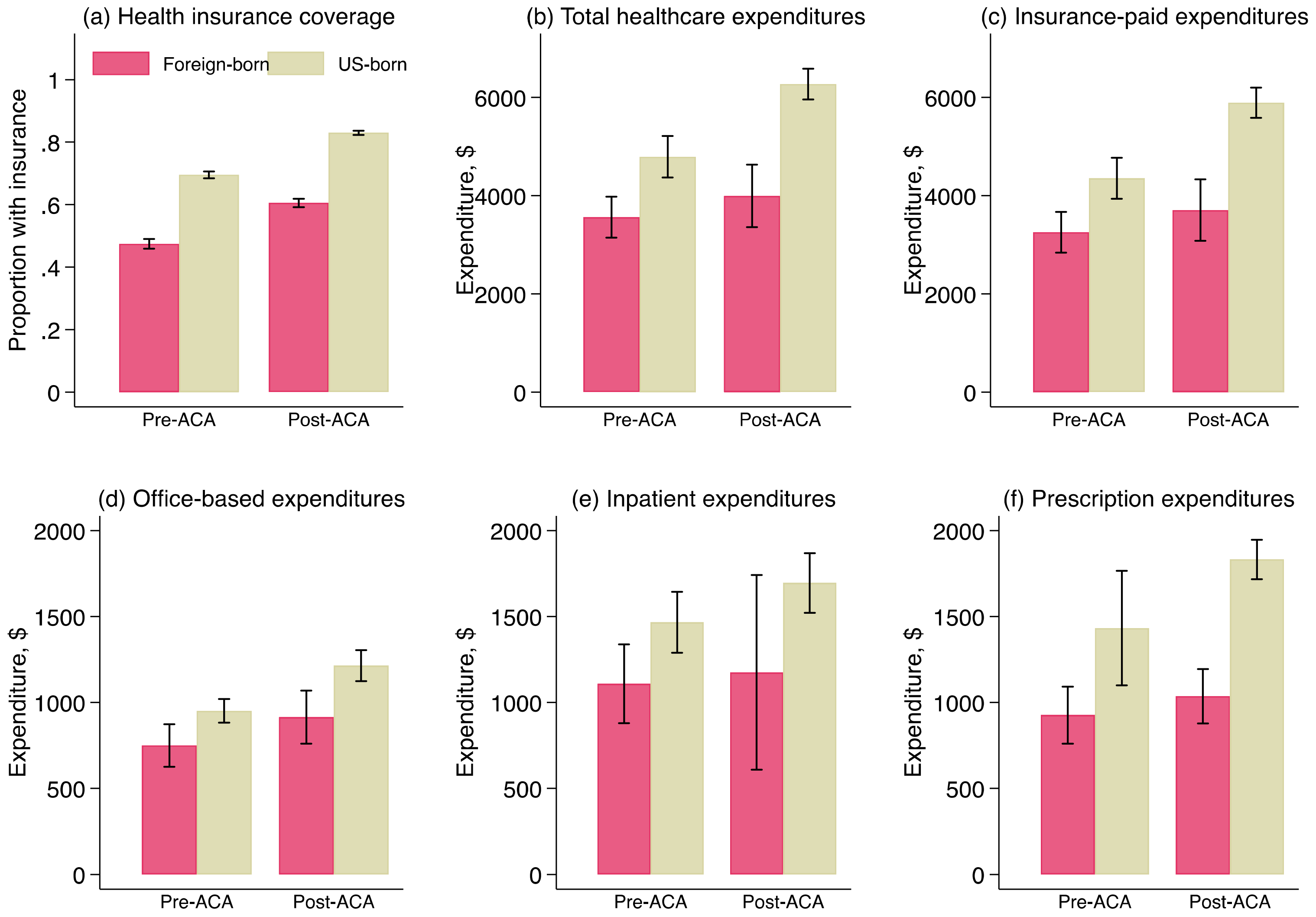

eFigure 1. Health Insurance Coverage and Annual Healthcare Expenditure by Nativity in the Pre-ACA (2011-2013) and Post-ACA (2014-2019) Periods, Adjusted for Whether Has Preexisting Chronic Condition. Note: Sample is restricted to adults aged 19-64 in households with incomes below 138% of the federal poverty threshold. Error bars are 95% confidence intervals.

© 2023 Kaushal N et al. *JAMA Network Open.*

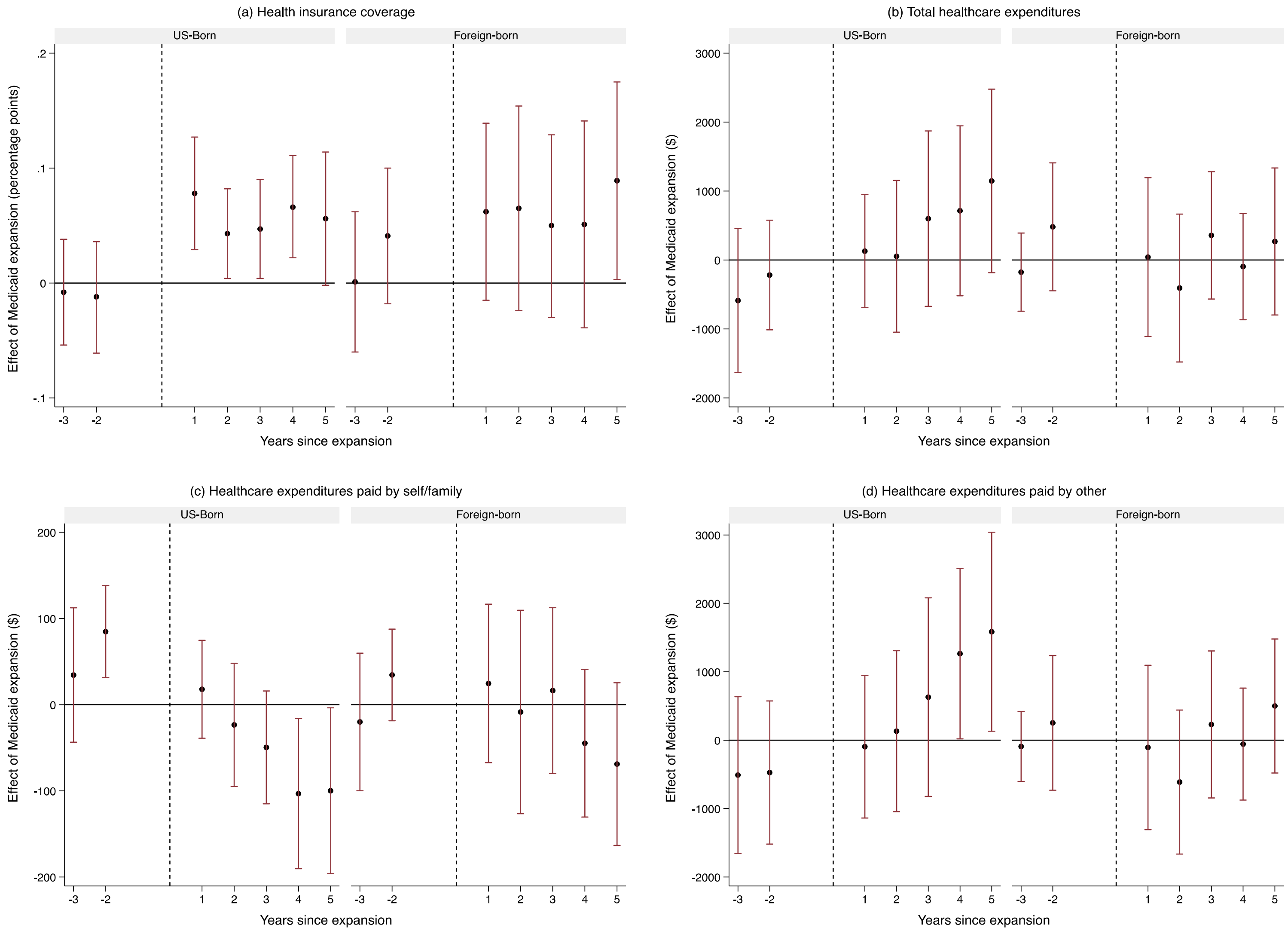

eFigure 2. Coefficient Estimates and 95% Confidence Intervals From Event Study of Medicaid Expansion by Nativity Status.
Figures display coefficients for the interaction of state Medicaid expansion with year relative to implementation. The year immediately prior to expansion is omitted as the reference year. Regressions control for age, sex, race/ethnicity, educational attainment, marital status, family size, number of children, state unemployment rate, per capita state GDP, state fixed effects, and year fixed effects. Robust standard errors are clustered by state of residence.

© 2023 Kaushal N et al. *JAMA Network Open.*

eTable 2. Estimates of the Effect of State Medicaid Expansion on Health Insurance Coverage and Annual Healthcare Expenditures of US-born and Foreign-Born Adults Aged 19-64, With Incomes Below 138% of the Federal Poverty Threshold, 2011-2019

	Insurance coverage		Total expenditures		Self-paid expenditures		Expenditures paid by others	
	(1)	(2)	(1)	(2)	(1)	(2)	(1)	(2)
**Panel 1: Whether outcome > 0**								
State Medicaid expansion	0.07***	0.07***	0.03***	0.03***	-0.01	-0.02	0.04***	0.04***
	(0.02)	(0.02)	(0.01)	(0.01)	(0.01)	(0.01)	(0.01)	(0.01)
Foreign-born	-0.19***	-0.18***	-0.10***	-0.08***	-0.07***	-0.05***	-0.13***	-0.10***
	(0.02)	(0.02)	(0.01)	(0.01)	(0.01)	(0.01)	(0.01)	(0.01)
State Medicaid expansion × Foreign-born	-0.02	-0.02	0.01	0.01	0.03*	0.03*	-0.01	-0.00
	(0.04)	(0.03)	(0.01)	(0.01)	(0.02)	(0.02)	(0.02)	(0.02)
Pre-ACA mean of whether outcome > 0	0.643	0.643	0.742	0.742	0.642	0.642	0.644	0.644
Number of Observations	43,961	43,942	43,961	43,942	43,961	43,942	43,961	43,942
**Panel 2: Log expenditure if expenditure > 0**								
State Medicaid expansion			0.07	0.08	-0.22***	-0.22***	0.08	0.08
			(0.05)	(0.05)	(0.07)	(0.07)	(0.05)	(0.05)
Foreign-born			-0.49***	-0.41***	-0.03	-0.00	-0.52***	-0.44***
			(0.05)	(0.05)	(0.05)	(0.05)	(0.06)	(0.06)
State Medicaid expansion × Foreign-born			-0.06	-0.04	0.13	0.14	-0.04	-0.02
			(0.06)	(0.07)	(0.10)	(0.10)	(0.07)	(0.08)
Pre-ACA mean of log expenditure			7.142	7.143	5.052	5.052	7.122	7.122
Number of Observations			31,231	31,224	25,282	25,276	27,994	27,988
Overall marginal effect of state Medicaid expansion on US-born			*690.77***	*660.13***	*-86.59****	*-86.55****	*802.22***	*745.34***
			(314.41)	*(296.69)*	*(24.70)*	*(24.41)*	*(326.35)*	*(308.43)*
Overall marginal effect of state Medicaid expansion on foreign-born			*170.80*	*266.21*	*-24.34*	*-22.74*	*215.97*	*308.02*
			(267.31)	*(313.25)*	*(27.91)*	*(29.57)*	*(282.47)*	*(336.50)*

Note: Sample is restricted to adults aged 19-64 in households with incomes below 138% of the federal poverty threshold. All models control for age, sex, race/ethnicity, educational attainment, marital status, family size, number of children, state unemployment rate, per capita state GDP, state fixed effects, and year fixed effects. Model 2 additionally controls for whether respondent had a pre-existing chronic condition. Marginal effects are derived from the combined first- and second-part models. Results are adjusted by MEPS sampling weights. Robust standard errors clustered on state of residence are in parentheses. *** p<0.01, ** p<0.05, * p<0.1.

© 2023 Kaushal N et al. *JAMA Network Open.*

eTable 3. Estimates of the Effect of State Medicaid Expansion on Annual Healthcare Expenditures, by Healthcare Type, of US-Born and Foreign-Born Adults Aged 19-64, 2011-2019

	Office-based expenditures		Inpatient expenditures		Prescription expenditures	
	(1)	(2)	(1)	(2)	(1)	(2)
**Panel 1: Whether any expenditure > 0**						
State Medicaid expansion	0.05***	0.05***	0.01	0.01	0.03***	0.02***
	(0.01)	(0.01)	(0.01)	(0.01)	(0.01)	(0.01)
Foreign-born	-0.09***	-0.07***	-0.04***	-0.03***	-0.11***	-0.08***
	(0.01)	(0.01)	(0.01)	(0.01)	(0.01)	(0.01)
State Medicaid expansion × Foreign-born	-0.02	-0.01	0.00	0.01	-0.00	0.00
	(0.01)	(0.01)	(0.01)	(0.01)	(0.01)	(0.01)
Pre-ACA mean of whether expenditure > 0	0.600	0.600	0.101	0.101	0.587	0.587
Number of observations	43,961	43,942	43,961	43,942	43,961	43,942
**Panel 2: Log expenditure if expenditure > 0**						
State Medicaid expansion	-0.00	0.00	0.01	0.01	0.12*	0.12*
	(0.04)	(0.04)	(0.10)	(0.11)	(0.06)	(0.06)
Foreign-born	-0.27***	-0.22***	-0.05	-0.04	-0.58***	-0.48***
	(0.04)	(0.03)	(0.15)	(0.15)	(0.08)	(0.07)
State Medicaid expansion × Foreign-born	-0.04	-0.02	0.02	0.02	-0.12	-0.09
	(0.04)	(0.04)	(0.14)	(0.14)	(0.10)	(0.09)
Pre-ACA mean of log expenditure	6.125	6.125	8.763	8.763	5.513	5.513
Number of observations	25,035	25,030	4,205	4,205	24,168	24,166
Overall marginal effect of state Medicaid expansion on US-born	*77.55*	*73.35*	*-45.23*	*-55.58*	*348.96***	*347.26***
	(49.73)	*(46.30)*	*(181.16)*	*(174.19)*	*(149.23)*	*(144.96)*
Overall marginal effect of state Medicaid expansion on foreign-born	*10.26*	*21.12*	*138.51*	*156.86*	*39.01*	*80.50*
	(39.84)	*(39.34)*	*(194.32)*	*(214.09)*	*(117.36)*	*(139.87)*

Note: See notes to eTable 2 for sample restriction and model specification. Marginal effects for inpatient expenditures are derived from models where the first part is a logit regression and the second part is a gamma regression with a log link. Robust standard errors clustered on state of residence are in parentheses.
*** p<0.01, ** p<0.05, * p<0.1.

© 2023 Kaushal N et al. *JAMA Network Open.*

eTable 4. Effect of State Medicaid Expansion on Healthcare Utilization of US-Born and Foreign-Born Adults Aged 19-64, 2011-2019

Number of/	Office-based visits		Outpatient facility visits		Emergency room visits		Hospital discharges	
	(1)	(2)	(1)	(2)	(1)	(2)	(1)	(2)
State Medicaid expansion	0.314***	0.301***	0.184**	0.181**	-0.010	-0.016	0.034	0.031
	(0.089)	(0.080)	(0.088)	(0.086)	(0.057)	(0.057)	(0.038)	(0.037)
Foreign-born	-0.674***	-0.503***	-0.143***	-0.094*	-0.486***	-0.409***	-0.201***	-0.165***
	(0.061)	(0.052)	(0.051)	(0.055)	(0.045)	(0.045)	(0.027)	(0.028)
State Medicaid expansion × Foreign-born	-0.132*	-0.098	-0.202***	-0.192***	0.085	0.101	0.024	0.031
	(0.074)	(0.076)	(0.066)	(0.067)	(0.061)	(0.064)	(0.050)	(0.052)
Pre-ACA mean of outcome variable	4.897	4.897	0.399	0.399	0.349	0.349	0.135	0.135
Number of observations	43,961	43,942	43,961	43,942	43,961	43,942	43,961	43,942

Number of/	Dental care visits		Home health provider days		Prescription medicine refills	
	(1)	(2)	(1)	(2)	(1)	(2)
State Medicaid expansion	0.178***	0.177***	0.080*	0.078*	0.241***	0.221***
	(0.055)	(0.055)	(0.046)	(0.046)	(0.070)	(0.059)
Foreign-born	-0.165***	-0.151**	-0.099***	-0.065**	-0.929***	-0.654***
	(0.058)	(0.057)	(0.031)	(0.029)	(0.083)	(0.073)
State Medicaid expansion × Foreign-born	0.031	0.034	-0.068	-0.061	-0.104	-0.049
	(0.071)	(0.071)	(0.053)	(0.054)	(0.084)	(0.088)
Pre-ACA mean of outcome variable	0.441	0.441	2.476	2.477	13.14	13.14
Number of observations	43,961	43,942	43,848	43,829	43,961	43,942

Note: Dependent variables are log-transformed in the regression analysis. See notes to eTable 2 for sample restriction and model specification. Robust standard errors clustered on state of residence are in parentheses. *** p<0.01, ** p<0.05, * p<0.1.

© 2023 Kaushal N et al. *JAMA Network Open.*

eTable 5. Effect of State Medicaid Expansion on (Dichotomized) Healthcare Utilization of US-Born and Foreign-Born Adults Aged 19-64, 2011-2019

	Had office-based visit		Had outpatient facility visit		Had emergency room visit		Had hospital discharge	
	(1)	(2)	(1)	(2)	(1)	(2)	(1)	(2)
State Medicaid expansion	0.048***	0.046***	0.035**	0.034**	-0.002	-0.003	0.008	0.008
	(0.013)	(0.012)	(0.016)	(0.016)	(0.011)	(0.011)	(0.008)	(0.008)
Foreign-born	-0.093***	-0.070***	-0.027***	-0.018*	-0.097***	-0.083***	-0.041***	-0.034***
	(0.010)	(0.009)	(0.010)	(0.011)	(0.009)	(0.009)	(0.006)	(0.006)
State Medicaid expansion × Foreign-born	-0.012	-0.007	-0.034***	-0.033**	0.018	0.021	0.005	0.006
	(0.012)	(0.013)	(0.012)	(0.013)	(0.012)	(0.013)	(0.010)	(0.011)
Pre-ACA mean of outcome variable	0.617	0.617	0.130	0.130	0.211	0.212	0.104	0.104
Number of observations	43,961	43,942	43,961	43,942	43,961	43,942	43,961	43,942

	Had dental care visit		Had home health provider day		Had prescription medicine refill	
	(1)	(2)	(1)	(2)	(1)	(2)
State Medicaid expansion	0.033***	0.033***	0.013*	0.013*	0.028***	0.026***
	(0.011)	(0.011)	(0.007)	(0.007)	(0.009)	(0.008)
Foreign-born	-0.034***	-0.031***	-0.012***	-0.007*	-0.114***	-0.082***
	(0.011)	(0.011)	(0.004)	(0.004)	(0.011)	(0.010)
State Medicaid expansion × Foreign-born	0.010	0.010	-0.011	-0.010	-0.004	0.003
	(0.013)	(0.013)	(0.007)	(0.007)	(0.013)	(0.013)
Pre-ACA mean of outcome variable	0.215	0.215	0.0303	0.0303	0.589	0.589
Number of observations	43,961	43,942	43,961	43,942	43,961	43,942

Note: See notes to eTable 2 for sample restriction and model specification. Robust standard errors clustered on state of residence are in parentheses. *** p<0.01, ** p<0.05, * p<0.1.

© 2023 Kaushal N et al. *JAMA Network Open.*

eTable 6. Estimates of the Effect of State Medicaid Expansion on Health Insurance Coverage and Annual Healthcare Expenditures of US-Born and Foreign-Born Adults in the US for 5+ Years Aged 19-64, 2011-2019

	Insurance coverage		Total expenditures		Self-paid expenditures		Expenditures paid by others	
	(1)	(2)	(1)	(2)	(1)	(2)	(1)	(2)
**Panel 1: Whether outcome > 0**								
State Medicaid expansion	0.07***	0.07***	0.04***	0.03***	-0.02	-0.02	0.04***	0.04***
	(0.02)	(0.02)	(0.01)	(0.01)	(0.01)	(0.01)	(0.01)	(0.01)
Foreign-born	-0.19***	-0.18***	-0.10***	-0.07***	-0.06***	-0.04***	-0.12***	-0.10***
	(0.03)	(0.03)	(0.01)	(0.01)	(0.01)	(0.01)	(0.01)	(0.01)
State Medicaid expansion × Foreign-born	-0.02	-0.02	0.00	0.01	0.03*	0.03**	-0.01	-0.00
	(0.04)	(0.04)	(0.01)	(0.01)	(0.01)	(0.02)	(0.02)	(0.02)
Pre-ACA mean of outcome variable	0.653	0.653	0.751	0.751	0.650	0.651	0.654	0.654
Number of observations	41,826	41,809	41,826	41,809	41,826	41,809	41,826	41,809
**Panel 2: Log expenditure if expenditure > 0**								
State Medicaid expansion			0.07	0.07	-0.22***	-0.22***	0.08	0.08
			(0.05)	(0.05)	(0.07)	(0.07)	(0.06)	(0.05)
Foreign-born			-0.53***	-0.45***	-0.03	0.00	-0.56***	-0.48***
			(0.05)	(0.05)	(0.05)	(0.05)	(0.06)	(0.06)
State Medicaid expansion × Foreign-born			-0.02	0.00	0.11	0.12	-0.01	0.02
			(0.07)	(0.08)	(0.10)	(0.10)	(0.08)	(0.09)
Pre-ACA mean of outcome variable			7.154	7.154	5.061	5.061	7.133	7.133
Number of observations			30,004	29,997	24,308	24,302	26,984	26,978
Overall marginal effect of state Medicaid expansion on US-born			*666.45***	*643.00***	*-86.77****	*-86.82****	*788.78***	*736.72***
			(319.15)	*(301.09)*	*(25.31)*	*(25.07)*	*(345.76)*	*(328.78)*
Overall marginal effect of state Medicaid expansion on foreign-born			*286.40*	*390.99*	*-29.11*	*-27.85*	*311.99*	*418.61*
			(282.76)	*(332.25)*	*(29.90)*	*(31.80)*	*(307.01)*	*(363.17)*

Note: Sample is restricted to adults aged 19-64 in households with incomes below 138% of FPL. Foreign-born sample restricted to those in the US for 5+ years. All models control for age, sex, race/ethnicity, educational attainment, marital status, family size, number of children, state unemployment rate, per capita state GDP, state fixed effects, and year fixed effects. Model 2 additionally controls for whether the respondent has a pre-existing chronic condition. Marginal effects are derived from the combined first- and second-part models. Robust standard errors clustered on state of residence are in parentheses. *** p<0.01, ** p<0.05, * p<0.1.

© 2023 Kaushal N et al. *JAMA Network Open.*

eTable 7. Estimates of the Effect of State Medicaid Expansion on Annual Healthcare Expenditures, by Healthcare Type, of US-Born Adults and Foreign-Born Adults in the US for 5+ Years Aged 19-64, 2011-2019

	Office-based expenditures		Inpatient expenditures		Prescription expenditures	
	(1)	(2)	(1)	(2)	(1)	(2)
**Panel 1: Whether any expenditure > 0**						
State Medicaid expansion	0.05***	0.05***	0.01	0.01	0.03***	0.03***
	(0.01)	(0.01)	(0.01)	(0.01)	(0.01)	(0.01)
Foreign-born	-0.08***	-0.06***	-0.04***	-0.04***	-0.11***	-0.08***
	(0.01)	(0.01)	(0.01)	(0.01)	(0.01)	(0.01)
State Medicaid expansion × Foreign-born	-0.02	-0.01	0.01	0.01	0.01	0.01
	(0.01)	(0.01)	(0.01)	(0.01)	(0.01)	(0.01)
Pre-ACA mean of whether expenditure > 0	0.608	0.608	0.103	0.103	0.597	0.598
Number of observations	41,826	41,809	41,826	41,809	41,826	41,809
**Panel 2: Log expenditure if expenditure > 0**						
State Medicaid expansion	-0.01	-0.00	0.01	0.01	0.12*	0.12**
	(0.05)	(0.04)	(0.10)	(0.11)	(0.06)	(0.06)
Foreign-born	-0.28***	-0.24***	-0.06	-0.06	-0.55***	-0.45***
	(0.04)	(0.03)	(0.13)	(0.14)	(0.08)	(0.07)
State Medicaid expansion × Foreign-born	-0.01	-0.00	0.07	0.08	-0.15	-0.13
	(0.05)	(0.05)	(0.13)	(0.13)	(0.09)	(0.09)
Pre-ACA mean of log expenditure	6.133	6.133	8.755	8.755	5.537	5.537
Number of observations	24,086	24,081	4,063	4,063	23,357	23,355
Overall marginal effect of state Medicaid expansion on US-born	*69.90*	*66.80*	*-45.89*	*-54.89*	*360.20***	*361.00***
	(51.39)	*(48.11)*	*(186.68)*	*(180.10)*	*(151.38)*	*(146.83)*
Overall marginal effect of state Medicaid expansion on foreign-born	*21.98*	*32.86*	*178.62*	*206.62*	*21.20*	*56.13*
	(46.21)	*(46.02)*	*(198.32)*	*(220.71)*	*(119.98)*	*(140.43)*

Note: See notes to eTable 6 for sample restriction and model specification. Marginal effects for inpatient expenditures are derived from models where the first part is a logit regression and the second part is a gamma regression with a log link. Robust standard errors clustered on state of residence are in parentheses. *** p<0.01, ** p<0.05, * p<0.1.

© 2023 Kaushal N et al. *JAMA Network Open.*

eTable 8. Estimates of the Effect of State Medicaid Expansion on Health Insurance Coverage and Annual Healthcare Expenditures of US-Born and Foreign-Born Adults (Alternate Specification Using State-Level Differences in Medicaid Eligibility for Foreign-Born Adults in the US for <5 Years), 2011-2019

	Insurance coverage		Total expenditures		Self-paid expenditures		Expenditures paid by others	
	(1)	(2)	(1)	(2)	(1)	(2)	(1)	(2)
**Panel 1: Whether outcome > 0**								
State Medicaid expansion	0.07***	0.07***	0.03***	0.03***	-0.01	-0.02	0.04***	0.04***
	(0.02)	(0.02)	(0.01)	(0.01)	(0.01)	(0.01)	(0.01)	(0.01)
Foreign-born	-0.18***	-0.18***	-0.10***	-0.08***	-0.07***	-0.05***	-0.13***	-0.10***
	(0.02)	(0.02)	(0.01)	(0.01)	(0.01)	(0.01)	(0.01)	(0.01)
State Medicaid expansion × Foreign-born	-0.02	-0.02	0.00	0.01	0.03*	0.03*	-0.01	-0.00
	(0.04)	(0.03)	(0.01)	(0.01)	(0.02)	(0.02)	(0.02)	(0.02)
Pre-ACA mean of outcome variable	0.643	0.643	0.742	0.742	0.642	0.642	0.644	0.644
Number of observations	43,961	43,942	43,961	43,942	43,961	43,942	43,961	43,942
**Panel 2: Log expenditure if expenditure > 0**								
State Medicaid expansion			0.06	0.07	-0.22***	-0.22***	0.07	0.07
			(0.05)	(0.05)	(0.07)	(0.07)	(0.05)	(0.05)
Foreign-born			-0.48***	-0.40***	-0.02	0.00	-0.51***	-0.43***
			(0.05)	(0.05)	(0.05)	(0.05)	(0.06)	(0.06)
State Medicaid expansion × Foreign-born			-0.08	-0.05	0.12	0.13	-0.07	-0.05
			(0.07)	(0.07)	(0.10)	(0.10)	(0.08)	(0.09)
Pre-ACA mean of outcome variable			7.142	7.143	5.052	5.052	7.122	7.122
Number of observations			31,231	31,224	25,282	25,276	27,994	27,988
Overall marginal effect of state Medicaid expansion on US-born			628.38**	599.32**	-87.65***	-87.51***	718.37**	665.80**
			(314.54)	(297.14)	(25.46)	(25.15)	(328.98)	(310.83)
Overall marginal effect of state Medicaid expansion on foreign-born			72.63	160.97	-28.16	-26.50	92.38	175.97
			(274.35)	(320.08)	(29.18)	(30.71)	(295.91)	(348.64)

Note: Sample is restricted to adults aged 19-64 in households with incomes below 138% of FPL. State Medicaid expansion policy variable for foreign-born adults in the US for <5 years is determined by state expansion status and Medicaid eligibility policy. All models control for age, sex, race/ethnicity, educational attainment, marital status, family size, number of children, state unemployment rate, per capita state GDP, state fixed effects, and year fixed effects. Model 2 additionally controls for whether the respondent has a pre-existing chronic condition. Marginal effects are derived from the combined first- and second-part models. Robust standard errors clustered on state of residence are in parentheses. *** p<0.01, ** p<0.05, * p<0.1.

© 2023 Kaushal N et al. *JAMA Network Open.*

eTable 9. Estimates of the Effect of State Medicaid Expansion on Annual Healthcare Expenditures, by Healthcare Type, of US-Born Adults and Foreign-Born Adults (Alternate Specification: Using State-Level Differences in Medicaid Eligibility for Foreign-Born Adults in the US for <5 Years), 2011-2019

	Office-based expenditures		Inpatient expenditures		Prescription expenditures	
	(1)	(2)	(1)	(2)	(1)	(2)
**Panel 1: Whether any expenditure > 0**						
State Medicaid expansion	0.06***	0.05***	0.00	0.00	0.03***	0.03***
	(0.01)	(0.01)	(0.01)	(0.01)	(0.01)	(0.01)
Foreign-born	-0.09***	-0.07***	-0.04***	-0.03***	-0.12***	-0.08***
	(0.01)	(0.01)	(0.01)	(0.01)	(0.01)	(0.01)
State Medicaid expansion × Foreign-born	-0.02	-0.01	0.00	0.00	0.00	0.01
	(0.01)	(0.01)	(0.01)	(0.01)	(0.01)	(0.01)
Pre-ACA mean of whether expenditure > 0	0.600	0.600	0.101	0.101	0.587	0.587
Number of observations	43,961	43,942	43,961	43,942	43,961	43,942
**Panel 2: Log expenditure if expenditure > 0**						
State Medicaid expansion	0.00	0.01	0.01	0.01	0.10	0.11
	(0.05)	(0.04)	(0.10)	(0.10)	(0.07)	(0.06)
Foreign-born	-0.27***	-0.22***	-0.05	-0.04	-0.56***	-0.46***
	(0.04)	(0.03)	(0.15)	(0.15)	(0.08)	(0.07)
State Medicaid expansion × Foreign-born	-0.03	-0.02	0.03	0.03	-0.16*	-0.14
	(0.04)	(0.04)	(0.14)	(0.14)	(0.09)	(0.09)
Pre-ACA mean of log expenditure	6.125	6.125	8.763	8.763	5.513	5.513
Number of observations	25,035	25,030	4,205	4,205	24,168	24,166
Overall marginal effect of state Medicaid expansion on US-born	84.49*	80.00*	-73.20	-83.36	308.63**	307.06**
	(51.30)	(47.95)	(178.12)	(171.23)	(152.54)	(148.92)
Overall marginal effect of state Medicaid expansion on foreign-born	24.71	36.52	80.23	93.67	-19.78	10.67
	(40.47)	(40.52)	(189.36)	(208.70)	(117.49)	(138.22)

Note: See notes to eTable 8 for sample restriction and model specification. Marginal effects for inpatient expenditures are derived from models where the first part is a logit regression and the second part is a gamma regression with a log link. Robust standard errors clustered on state of residence are in parentheses. *** p<0.01, ** p<0.05, * p<0.1.

© 2023 Kaushal N et al. *JAMA Network Open.*

eTable 10. Estimates of the Effect of State Medicaid Expansion on Health Insurance Coverage and Annual Healthcare Expenditures of US-Born and Foreign-Born Adults Aged 19-64, Excluding Early and Late Expansion States, 2011-2019

	Insurance coverage		Total expenditures		Self-paid expenditures		Expenditures paid by others	
	(1)	(2)	(1)	(2)	(1)	(2)	(1)	(2)
**Panel 1: Whether outcome > 0**								
State Medicaid expansion	0.07***	0.07***	0.05***	0.04***	-0.00	-0.01	0.05***	0.04***
	(0.02)	(0.02)	(0.01)	(0.01)	(0.02)	(0.01)	(0.01)	(0.01)
Foreign-born	-0.18***	-0.17***	-0.10***	-0.08***	-0.07***	-0.05***	-0.13***	-0.10***
	(0.02)	(0.02)	(0.01)	(0.01)	(0.01)	(0.01)	(0.01)	(0.01)
State Medicaid expansion × Foreign-born	-0.01	-0.01	0.00	0.00	0.02	0.02	-0.00	-0.00
	(0.04)	(0.04)	(0.01)	(0.01)	(0.02)	(0.02)	(0.01)	(0.01)
Pre-ACA mean of whether outcome > 0	0.626	0.626	0.733	0.733	0.638	0.638	0.632	0.632
Number of observations	36,778	36,763	36,778	36,763	36,778	36,763	36,778	36,763
**Panel 2: Log expenditure if expenditure > 0**								
State Medicaid expansion			0.10	0.09	-0.28***	-0.28***	0.14*	0.14*
			(0.06)	(0.06)	(0.07)	(0.07)	(0.07)	(0.07)
Foreign-born			-0.51***	-0.42***	-0.01	0.02	-0.55***	-0.47***
			(0.05)	(0.05)	(0.05)	(0.05)	(0.06)	(0.06)
State Medicaid expansion × Foreign-born			-0.05	-0.03	0.20*	0.20*	-0.06	-0.05
			(0.08)	(0.08)	(0.10)	(0.10)	(0.07)	(0.08)
Pre-ACA mean of log expenditure			7.149	7.149	5.116	5.116	7.128	7.128
Number of observations			25,824	25,817	20,993	20,987	23,036	23,030
Overall marginal effect of state Medicaid expansion on US-born			*937.83***	*823.95***	*-103.01****	*-105.83****	*1,264.63****	*1,135.86****
			(392.91)	*(389.39)*	*(27.47)*	*(27.28)*	*(437.73)*	*(429.06)*
Overall marginal effect of state Medicaid expansion on foreign-born			*336.60*	*366.61*	*-17.09*	*-20.34*	*378.13*	*421.67*
			(339.14)	*(391.22)*	*(35.80)*	*(39.22)*	*(343.43)*	*(394.78)*

Note: Sample is restricted to adults aged 19-64 in households with incomes below 138% of the federal poverty threshold living in states that expanded in early 2014 or that did not expand during the study period. All models control for age, sex, race/ethnicity, educational attainment, marital status, family size, number of children, state unemployment rate, per capita state GDP, state fixed effects, and year fixed effects. Model 2 additionally controls for whether the respondent had a pre-existing chronic condition. Marginal effects are derived from the combined first- and second-part models. Robust standard errors clustered on state of residence are in parentheses. *** p<0.01, ** p<0.05, * p<0.1.

© 2023 Kaushal N et al. *JAMA Network Open.*

eTable 11. Estimates of the Effect of State Medicaid Expansion on Annual Healthcare Expenditures, by Healthcare Type, of US-Born and Foreign-Born Adults Aged 19-64, Excluding Early and Late Expansion States, 2011-2019

	Office-based expenditures		Inpatient expenditures		Prescription expenditures	
	(1)	(2)	(1)	(2)	(1)	(2)
**Panel 1: Whether any expenditure > 0**						
State Medicaid expansion	0.07***	0.06***	0.01*	0.01*	0.04**	0.03**
	(0.02)	(0.01)	(0.01)	(0.01)	(0.01)	(0.01)
Foreign-born	-0.09***	-0.07***	-0.04***	-0.04***	-0.12***	-0.08***
	(0.01)	(0.01)	(0.01)	(0.01)	(0.01)	(0.01)
State Medicaid expansion × Foreign-born	-0.03**	-0.02*	-0.00	-0.00	0.00	0.00
	(0.01)	(0.01)	(0.01)	(0.01)	(0.01)	(0.01)
Pre-ACA mean of whether expenditure > 0	0.594	0.594	0.100	0.100	0.583	0.583
Number of observations	36,778	36,763	36,778	36,763	36,778	36,763
**Panel 2: Log expenditure if expenditure > 0**						
State Medicaid expansion	0.02	0.01	-0.01	-0.01	0.17**	0.16**
	(0.06)	(0.06)	(0.12)	(0.12)	(0.08)	(0.08)
Foreign-born	-0.28***	-0.22***	-0.11	-0.11	-0.61***	-0.51***
	(0.04)	(0.04)	(0.15)	(0.15)	(0.10)	(0.09)
State Medicaid expansion × Foreign-born	0.02	0.02	0.03	0.03	-0.06	-0.05
	(0.04)	(0.04)	(0.13)	(0.13)	(0.10)	(0.10)
Pre-ACA mean of log expenditure	6.145	6.145	8.771	8.771	5.527	5.527
Number of observations	20,717	20,712	3,406	3,406	20,052	20,050
Overall marginal effect of state Medicaid expansion on US-born	*122.63**	*105.70**	*79.88*	*62.89*	*504.38****	*488.41***
	(63.40)	*(61.16)*	*(202.86)*	*(197.28)*	*(182.67)*	*(192.29)*
Overall marginal effect of state Medicaid expansion on foreign-born	*70.50*	*70.98*	*89.90*	*91.00*	*178.54*	*207.22*
	(45.11)	*(46.65)*	*(216.03)*	*(233.39)*	*(126.81)*	*(156.69)*

Note: See notes to eTable 10 for sample restriction and model specification. Marginal effects for inpatient expenditures are derived from models where the first part is a logit regression and the second part is a gamma regression with a log link. Robust standard errors clustered on state of residence are in parentheses. *** p<0.01, ** p<0.05, * p<0.1.

© 2023 Kaushal N et al. *JAMA Network Open.*

eTable 12. Estimates of the Effect of State Medicaid Expansion on Health Insurance Coverage and Annual Healthcare Expenditures of US-Born and Foreign-Born Adults Aged 19-64 With Incomes Below 100% of FPL, 2011-2019

	Insurance coverage		Total expenditures		Self-paid expenditures		Expenditures paid by others	
	(1)	(2)	(1)	(2)	(1)	(2)	(1)	(2)
**Panel 1: Whether outcome > 0**								
State Medicaid expansion	0.06***	0.06***	0.02***	0.02***	-0.01	-0.02	0.03**	0.03**
	(0.02)	(0.02)	(0.01)	(0.01)	(0.01)	(0.01)	(0.01)	(0.01)
Foreign-born	-0.18***	-0.17***	-0.09***	-0.07***	-0.05***	-0.03**	-0.12***	-0.10***
	(0.03)	(0.02)	(0.01)	(0.01)	(0.01)	(0.01)	(0.01)	(0.01)
State Medicaid expansion × Foreign-born	-0.02	-0.01	0.01	0.01	0.01	0.02	0.00	0.01
	(0.04)	(0.04)	(0.02)	(0.02)	(0.02)	(0.02)	(0.02)	(0.02)
Pre-ACA mean of whether outcome > 0	0.646	0.646	0.753	0.753	0.642	0.642	0.660	0.660
Number of observations	30,266	30,257	30,266	30,257	30,266	30,257	30,266	30,257
**Panel 2: Log expenditure if expenditure > 0**								
State Medicaid expansion			0.13*	0.13**	-0.22***	-0.22***	0.15**	0.16**
			(0.06)	(0.06)	(0.07)	(0.07)	(0.07)	(0.07)
Foreign-born			-0.48***	-0.40***	-0.03	-0.00	-0.49***	-0.40***
			(0.06)	(0.06)	(0.06)	(0.06)	(0.07)	(0.07)
State Medicaid expansion × Foreign-born			-0.07	-0.05	0.26**	0.26**	-0.13	-0.10
			(0.09)	(0.09)	(0.12)	(0.12)	(0.10)	(0.11)
Pre-ACA mean of log expenditure			7.192	7.192	4.971	4.971	7.182	7.182
Number of observations			21,720	21,717	17,301	17,299	19,595	19,593
Overall marginal effect of state Medicaid expansion on US-born			*1,035.58***	*999.91***	*-85.05****	*-84.83****	*1,267.95****	*1,213.30****
			(429.90)	*(418.21)*	*(26.30)*	*(26.26)*	*(435.01)*	*(421.77)*
Overall marginal effect of state Medicaid expansion on foreign-born			*300.19*	*419.83*	*12.16*	*16.47*	*182.55*	*287.35*
			(366.58)	*(427.88)*	*(37.24)*	*(39.03)*	*(395.90)*	*(461.60)*

Note: Sample is restricted to adults aged 19-64 in households with incomes below 100% of the federal poverty threshold. All models control for age, sex, race/ethnicity, educational attainment, marital status, family size, number of children, state unemployment rate, per capita state GDP, state fixed effects, and year fixed effects. Model 2 additionally controls for whether the respondent had a pre-existing chronic condition. Marginal effects are derived from the combined first- and second-part models. Robust standard errors clustered on state of residence are in parentheses. *** p<0.01, ** p<0.05, * p<0.1.

© 2023 Kaushal N et al. *JAMA Network Open.*

eTable 13. Estimates of the Effect of State Medicaid Expansion on Annual Healthcare Expenditures, by Healthcare Type, of US-Born and Foreign-Born Adults Aged 19-64 With Incomes Below 100% of FPL, 2011-2019

	Office-based expenditures		Inpatient expenditures		Prescription expenditures	
	(1)	(2)	(1)	(2)	(1)	(2)
**Panel 1: Whether any expenditure > 0**						
State Medicaid expansion	0.05***	0.05***	0.01	0.01	0.02*	0.02
	(0.01)	(0.01)	(0.01)	(0.01)	(0.01)	(0.01)
Foreign-born	-0.08***	-0.06***	-0.04***	-0.03***	-0.11***	-0.08***
	(0.01)	(0.01)	(0.01)	(0.01)	(0.01)	(0.01)
State Medicaid expansion × Foreign-born	-0.02	-0.01	0.00	0.00	-0.01	-0.00
	(0.01)	(0.01)	(0.02)	(0.02)	(0.02)	(0.02)
Pre-ACA mean of whether expenditure > 0	0.611	0.611	0.109	0.109	0.605	0.605
Number of observations	30,266	30,257	30,266	30,257	30,266	30,257
**Panel 2: Log expenditure if expenditure > 0**						
State Medicaid expansion	0.00	0.00	0.10	0.11	0.12	0.12
	(0.05)	(0.06)	(0.13)	(0.13)	(0.08)	(0.08)
Foreign-born	-0.27***	-0.22***	-0.07	-0.05	-0.58***	-0.47***
	(0.05)	(0.05)	(0.17)	(0.17)	(0.11)	(0.10)
State Medicaid expansion × Foreign-born	-0.05	-0.04	0.13	0.13	-0.11	-0.09
	(0.05)	(0.05)	(0.14)	(0.14)	(0.12)	(0.12)
Pre-ACA mean of log expenditure	6.134	6.134	8.744	8.744	5.545	5.545
Number of observations	17,457	17,456	3,189	3,189	16,991	16,990
Overall marginal effect of state Medicaid expansion on US-born	*73.15*	*69.03*	*41.20*	*36.26*	*382.89**	*389.78**
	(59.53)	*(59.00)*	*(247.72)*	*(241.01)*	*(208.80)*	*(207.44)*
Overall marginal effect of state Medicaid expansion on foreign-born	*-2.50*	*8.34*	*224.30*	*255.02*	*22.89*	*76.44*
	(44.53)	*(46.56)*	*(264.10)*	*(291.90)*	*(171.56)*	*(207.42)*

Note: See notes to eTable 3 for sample restrictions and model specification. Marginal effects for inpatient expenditures are derived from models where the first part is a logit regression and the second part is a gamma regression with a log link. Robust standard errors clustered on state of residence are in parentheses.
*** p<0.01, ** p<0.05, * p<0.1.

© 2023 Kaushal N et al. *JAMA Network Open.*

eTable 14. Estimates of the Effect of State Medicaid Expansion on Health Insurance Coverage and Annual Healthcare Expenditures of US-Born and Foreign-Born Adults Aged 19-64, With Additional Adjustment for Employment Status, 2011-2019

	Insurance coverage		Total expenditures		Self-paid expenditures		Expenditures paid by others	
	(1)	(2)	(1)	(2)	(1)	(2)	(1)	(2)
**Panel 1: Whether outcome > 0**								
State Medicaid expansion	0.07***	0.07***	0.03***	0.03***	-0.02	-0.02	0.04***	0.04***
	(0.02)	(0.02)	(0.01)	(0.01)	(0.01)	(0.01)	(0.01)	(0.01)
Foreign-born	-0.18***	-0.17***	-0.10***	-0.08***	-0.06***	-0.04***	-0.12***	-0.10***
	(0.02)	(0.02)	(0.01)	(0.01)	(0.01)	(0.01)	(0.01)	(0.01)
State Medicaid expansion × Foreign-born	-0.02	-0.02	0.01	0.01	0.03*	0.03**	-0.01	-0.00
	(0.04)	(0.04)	(0.01)	(0.01)	(0.01)	(0.02)	(0.02)	(0.02)
Pre-ACA mean of whether outcome > 0	0.645	0.645	0.746	0.746	0.646	0.646	0.648	0.648
Number of Observations	42,970	42,961	42,970	42,961	42,970	42,961	42,970	42,961
**Panel 2: Log expenditure if expenditure > 0**								
State Medicaid expansion			0.08	0.08*	-0.21***	-0.21***	0.08	0.08
			(0.05)	(0.05)	(0.07)	(0.07)	(0.06)	(0.05)
Foreign-born			-0.44***	-0.36***	-0.05	-0.02	-0.47***	-0.40***
			(0.05)	(0.05)	(0.05)	(0.05)	(0.05)	(0.05)
State Medicaid expansion × Foreign-born			-0.07	-0.05	0.12	0.13	-0.04	-0.02
			(0.07)	(0.07)	(0.10)	(0.10)	(0.07)	(0.08)
Pre-ACA mean of log expenditure			7.145	7.145	5.054	5.054	7.124	7.124
Number of Observations			30,738	30,735	24,887	24,884	27,556	27,553
Overall marginal effect of state Medicaid expansion on US-born			*730.09***	*696.35***	*-85.72****	*-86.07****	*804.86***	*744.65***
			(312.59)	*(298.11)*	*(24.97)*	*(24.64)*	*(339.69)*	*(327.52)*
Overall marginal effect of state Medicaid expansion on foreign-born			*182.67*	*276.69*	*-25.54*	*-23.82*	*239.83*	*328.74*
			(295.45)	*(339.83)*	*(28.18)*	*(29.88)*	*(306.77)*	*(359.05)*

Note: Sample is restricted to adults aged 19-64 in households with incomes below 138% of the federal poverty threshold. All models control for age, sex, race/ethnicity, educational attainment, marital status, family size, number of children, employment status, state unemployment rate, per capita state GDP, state fixed effects, and year fixed effects. Model 2 additionally controls for whether respondent had a pre-existing chronic condition. Marginal effects are derived from the combined first- and second-part models. Results are adjusted by MEPS sampling weights. Robust standard errors clustered on state of residence are in parentheses. *** p<0.01, ** p<0.05, * p<0.1.

© 2023 Kaushal N et al. *JAMA Network Open.*

eTable 15. Estimates of the Effect of State Medicaid Expansion on Health Insurance Coverage and Annual Healthcare Expenditures of US-Born and Foreign-Born Adults Aged 19-64 Using MEPS 2011-2018

	Insurance coverage		Total expenditures		Self-paid expenditures		Expenditures paid by others	
Sample Household Income:	<138% FPL	<100% FPL	<138% FPL	<100% FPL	<138% FPL	<100% FPL	<138% FPL	<100% FPL
**Panel 1: Whether outcome > 0**								
State Medicaid expansion	0.07***	0.06***	0.03***	0.02**	-0.02	-0.02	0.04***	0.03**
	(0.02)	(0.02)	(0.01)	(0.01)	(0.01)	(0.01)	(0.01)	(0.01)
Foreign-born	-0.18***	-0.17***	-0.08***	-0.07***	-0.05***	-0.03**	-0.10***	-0.09***
	(0.02)	(0.03)	(0.01)	(0.01)	(0.01)	(0.01)	(0.01)	(0.01)
State Medicaid expansion × Foreign-born	-0.03	-0.02	0.02	0.01	0.04**	0.02	0.00	0.01
	(0.04)	(0.04)	(0.02)	(0.02)	(0.02)	(0.02)	(0.02)	(0.03)
Pre-ACA mean of whether outcome > 0	0.643	0.646	0.742	0.753	0.642	0.642	0.644	0.660
Number of observations	40,719	28,008	40,719	28,008	40,719	28,008	40,719	28,008
**Panel 2: Log expenditure if expenditure > 0**								
State Medicaid expansion			0.08	0.14**	-0.20***	-0.22***	0.08	0.17**
			(0.05)	(0.06)	(0.07)	(0.08)	(0.06)	(0.07)
Foreign-born			-0.40***	-0.37***	-0.03	-0.02	-0.44***	-0.38***
			(0.05)	(0.06)	(0.05)	(0.07)	(0.05)	(0.06)
State Medicaid expansion × Foreign-born			-0.09	-0.11	0.12	0.23*	-0.09	-0.18
			(0.07)	(0.09)	(0.10)	(0.12)	(0.09)	(0.12)
Pre-ACA mean of log expenditure			7.143	7.192	5.052	4.971	7.122	7.182
Number of observations			28,779	20,005	23,341	15,962	25,693	17,984
Overall marginal effect of state Medicaid expansion on US-born			*632.97***	*1,020.88***	*-81.68****	*-83.20****	*699.42***	*1,255.45****
			(287.15)	*(408.44)*	*(24.28)*	*(27.55)*	*(326.62)*	*(429.52)*
Overall marginal effect of state Medicaid expansion on foreign-born			*73.18*	*204.36*	*-16.92*	*10.00*	*73.83*	*55.48*
			(288.86)	*(400.98)*	*(28.75)*	*(38.91)*	*(340.33)*	*(480.03)*

Note: Sample is restricted to adults aged 19-64 in households with incomes below 138% (column 1) and 100% (column 2) of the federal poverty threshold. All models control for age, sex, race/ethnicity, educational attainment, marital status, family size, number of children, state unemployment rate, per capita state GDP, state fixed effects, year fixed effects, and whether the respondent had a pre-existing chronic condition. Marginal effects are derived from the combined first- and second-part models. Robust standard errors clustered on state of residence are in parentheses. *** p<0.01, ** p<0.05, * p<0.1.

© 2023 Kaushal N et al. *JAMA Network Open.*

eTable 16. Estimates of the Effect of State Medicaid Expansion on Health Insurance Coverage and Annual Healthcare Expenditures of US-Born and Foreign-Born Adults Aged 19-64 Using MEPS 2011-2020

	Insurance coverage		Total expenditures		Self-paid expenditures		Expenditures paid by others	
Sample Household Income:	<138% FPL	<100% FPL	<138% FPL	<100% FPL	<138% FPL	<100% FPL	<138% FPL	<100% FPL
**Panel 1: Whether outcome > 0**								
State Medicaid expansion	0.06***	0.05**	0.03***	0.02***	-0.02	-0.02	0.04***	0.02**
	(0.02)	(0.02)	(0.01)	(0.01)	(0.01)	(0.01)	(0.01)	(0.01)
Foreign-born	-0.17***	-0.18***	-0.08***	-0.08***	-0.05***	-0.04**	-0.11***	-0.10***
	(0.02)	(0.03)	(0.01)	(0.01)	(0.01)	(0.02)	(0.01)	(0.01)
State Medicaid expansion × Foreign-born	-0.01	-0.00	0.01	0.02	0.03*	0.03	0.00	0.01
	(0.03)	(0.04)	(0.01)	(0.02)	(0.02)	(0.02)	(0.02)	(0.02)
Pre-ACA mean of whether outcome > 0	0.643	0.646	0.742	0.753	0.642	0.642	0.644	0.660
Number of observations	47,111	32,466	47,111	32,466	47,111	32,466	47,111	32,466
**Panel 2: Log expenditure if expenditure > 0**								
State Medicaid expansion			0.04	0.08	-0.23***	-0.26***	0.04	0.12*
			(0.05)	(0.06)	(0.06)	(0.07)	(0.05)	(0.07)
Foreign-born			-0.41***	-0.40***	-0.02	-0.02	-0.44***	-0.39***
			(0.05)	(0.06)	(0.05)	(0.06)	(0.05)	(0.06)
State Medicaid expansion × Foreign-born			-0.02	-0.04	0.17*	0.29**	-0.02	-0.09
			(0.07)	(0.09)	(0.10)	(0.12)	(0.08)	(0.10)
Pre-ACA mean of log expenditure			7.143	7.192	5.052	4.971	7.122	7.182
Number of observations			33,568	23,336	27,129	18,583	30,163	21,100
Overall marginal effect of state Medicaid expansion on US-born			*412.92*	*675.65*	*-94.72****	*-102.81****	*499.77**	*981.18***
			(283.74)	*(426.23)*	*(24.21)*	*(27.19)*	*(296.95)*	*(429.87)*
Overall marginal effect of state Medicaid expansion on foreign-born			*182.66*	*272.00*	*-16.26*	*15.90*	*160.61*	*209.04*
			(316.73)	*(428.11)*	*(29.88)*	*(40.65)*	*(348.97)*	*(453.90)*

Note: Sample is restricted to adults aged 19-64 in households with incomes below 138% (column 1) and 100% (column 2) of the federal poverty threshold. All models control for age, sex, race/ethnicity, educational attainment, marital status, family size, number of children, state unemployment rate, per capita state GDP, state fixed effects, year fixed effects, and whether the respondent had a pre-existing chronic condition. Marginal effects are derived from the combined first- and second-part models. Robust standard errors clustered on state of residence are in parentheses. *** p<0.01, ** p<0.05, * p<0.1.

© 2023 Kaushal N et al. *JAMA Network Open.*
