# Supplementary material for: Cost of Public Health Insurance for US-Born and Immigrant Adults
Source: JAMA Netw Open. 2023 Sep 15;6(9):e2334008. doi: 10.1001/jamanetworkopen.2023.34008 (PMC10504616; doi:10.1001/jamanetworkopen.2023.34008)
Supplement: Supplement 2. — Data Sharing Statement [file jamanetwopen-e2334008-s002.pdf]

Data Sharing Statement

Kaushal. Cost of Public Health Insurance for US-Born and Immigrant Adults. *JAMA Netw Open*. Published September 15, 2023. doi:10.1001/jamanetworkopen.2023.34008

Data
Data available: No

Additional Information
Explanation for why data not available: Restricted MEPS data files can be made available upon request to the Agency for Healthcare Research and Quality (AHRQ) (https://meps.ahrq.gov/mepsweb/) and can only be accessed at an AHRQ data center or a Federal Statistical Research Data Center. Public use MEPS data files and codebooks are publicly available through AHRQ (https://meps.ahrq.gov/mepsweb/).
